# During FeS cluster biogenesis, ferredoxin and frataxin use overlapping binding sites on yeast cysteine desulfurase Nfs1

**DOI:** 10.1016/j.jbc.2022.101570

**Published:** 2022-01-11

**Authors:** Marta A. Uzarska, Igor Grochowina, Joanna Soldek, Marcin Jelen, Brenda Schilke, Jaroslaw Marszalek, Elizabeth A. Craig, Rafal Dutkiewicz

**Affiliations:** 1 Intercollegiate Faculty of Biotechnology, University of Gdansk and Medical University of Gdansk, Gdansk, Poland; 2 Department of Biochemistry, University of Wisconsin - Madison, Madison, Wisconsin, USA

**Keywords:** iron–sulfur protein, mitochondria, protein–protein interactions, protein complex, protein evolution, ACP, acyl carrier protein, ACP_Ec_, *E. coli* ACP, BLI, biolayer interferometry, FeS, iron–sulfur, GST, glutathione-*S*-transferase, HMM, hidden Markov model, Isu1_Ct_, Isu1 ortholog of *C. thermophilum*, Isu1_Sc_, Isu1 ortholog of *S. cerevisiae*, *k*_*d*_, dissociation rate, *K*_*D*_, equilibrium dissociation constant, mdeg, millidegrees, MS, mass spectrometry, NIA, Nfs1–Isd11–ACP, OMA, *o*rthologous *ma*trix, PDB, Protein Data Bank, Yah1^GST^, Yah1-GST fusion protein, Yfh1^GST^, Yfh1-GST fusion protein

## Abstract

In mitochondria, cysteine desulfurase (Nfs1) plays a central role in the biosynthesis of iron–sulfur (FeS) clusters, cofactors critical for activity of many cellular proteins. Nfs1 functions both as a sulfur donor for cluster assembly and as a binding platform for other proteins functioning in the process. These include not only the dedicated scaffold protein (Isu1) on which FeS clusters are synthesized but also accessory FeS cluster biogenesis proteins frataxin (Yfh1) and ferredoxin (Yah1). Yfh1 has been shown to activate cysteine desulfurase enzymatic activity, whereas Yah1 supplies electrons for the persulfide reduction. While Yfh1 interaction with Nfs1 is well understood, the Yah1–Nfs1 interaction is not. Here, based on the results of biochemical experiments involving purified WT and variant proteins, we report that in *Saccharomyces cerevisiae*, Yah1 and Yfh1 share an evolutionary conserved interaction site on Nfs1. Consistent with this notion, Yah1 and Yfh1 can each displace the other from Nfs1 but are inefficient competitors when a variant with an altered interaction site is used. Thus, the binding mode of Yah1 and Yfh1 interacting with Nfs1 in mitochondria of *S. cerevisiae* resembles the mutually exclusive binding of ferredoxin and frataxin with cysteine desulfurase reported for the bacterial FeS cluster assembly system. Our findings are consistent with the generally accepted scenario that the mitochondrial FeS cluster assembly system was inherited from bacterial ancestors of mitochondria.

Iron–sulfur (FeS) clusters are inorganic cofactors required for the activity of many proteins including enzymes across all forms of life (1, 2). Such proteins function in a variety of cellular processes, including oxidative respiration, metabolism, biosynthesis of cofactors, RNA and DNA transactions, and regulation of gene expression (3, 4). In eukaryotes, mitochondria, which inherited their FeS cluster biogenesis pathway from bacterial ancestors (5), play a key role in this process. Because the mitochondrial FeS cluster assembly system of *Saccharomyces cerevisiae* is the focus of this report, we use the protein nomenclature of this organism throughout.

FeS clusters are assembled on a dedicated scaffold protein (Isu1) (3). The cysteine desulfurase Nfs1 plays a key role as the sulfur donor, whereas the source of ferrous iron needed for cluster assembly remains unknown. Nfs1, a pyridoxal phosphate–dependent enzyme, generates persulfide from l-cysteine and delivers it to Isu1. Nfs1 also serves as a binding platform for proteins involved in cluster assembly, such as Isu1, frataxin Yfh1, and ferredoxin Yah1 (6, 7, 8, 9, 10). Yfh1, which in humans is implicated in the neurological disease Friedreich's ataxia characterized by impairment of FeS biogenesis and iron metabolism, facilitates persulfide transfer from Nfs1 to Isu1 (11, 12, 13, 14, 15, 16, 17, 18, 19). Yah1 harbors a [2Fe–2S] cluster able to accept or discharge electrons depending on its oxidation state; it, along with its cognate reductase Arh1, delivers electrons provided by NADPH to reduce the persulfide on Isu1 to sulfide, the form present in its FeS cluster (19, 20, 21, 22, 23, 24, 25).

Bacterial Nfs1 (IscS) functions as a homodimer, forming an elongated structure stabilized by a large binding interface (7, 8). Bacterial Isu1 (IscU) binds to opposite ends of the IscS dimer, forming an IscS–IscU heterotetramer of α_2_β_2_ stoichiometry (Fig. 1*A*). Bacterial frataxin (CyaY) and ferredoxin (Fdx) interact with the IscS–IscU complex in a mutually exclusive manner, utilizing overlapping binding sites localized in the cleft between the two IscS protomers next to the IscU (22, 26, 27).

**Figure 1:**
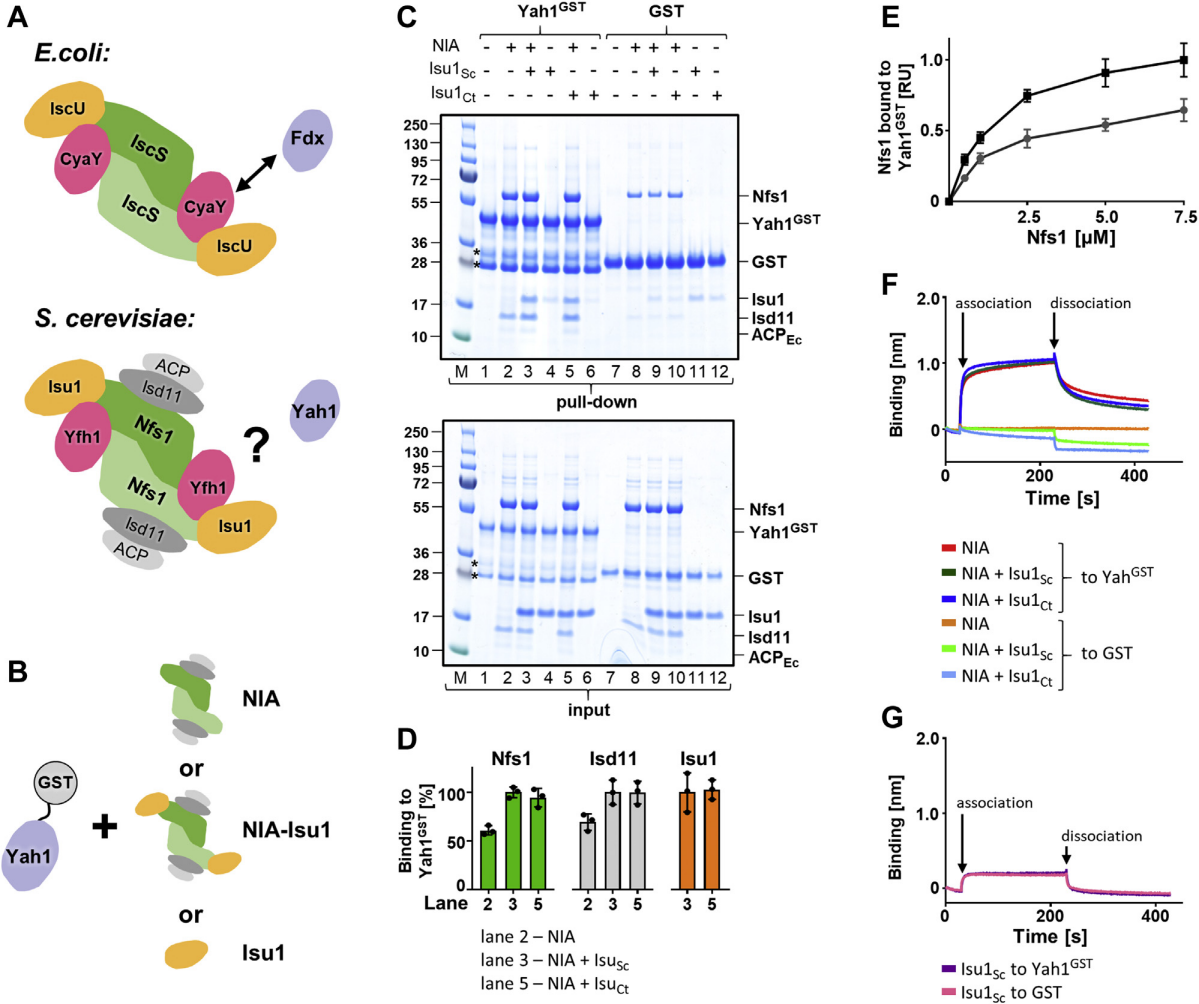
**Yah1 binding to NIA complex is independent of Isu1.***A*, cartoon of cysteine desulfurase complex involved in FeS cluster biogenesis in *Escherichia coli* and *Saccharomyces cerevisiae*. In both cases, cysteine desulfurase (IscS–Nfs1) forms a dimer; however, in *S. cerevisiae*, Nfs1 forms a stable complex with the accessory protein Isd11, which binds acyl carrier protein (ACP) together forming the NIA complex protomer. FeS cluster scaffold (IscU–Isu1) and frataxin (CyaY–Yfh1) interact with IscS–NIA at the conserved sites. In *E. coli*, ferredoxin Fdx competes with frataxin CyaY for overlapping binding sites on IscS, whereas in *S. cerevisiae*, the mode of ferredoxin Yah1 binding to the NIA complex is unresolved. *B*, Yah1^GST^ was used to test ferredoxin interaction with purified NIA, NIA–Isu1, and Isu1 using both pull-down and biolayer interferometry (BLI) assays. *C*, *top*, Yah1^GST^ (2.5 μM) or GST (2.5 μM) was incubated with NIA (5 μM), Isu1_Sc_ (7.5 μM), or Isu1_Ct_ (7.5 μM), as indicated. Glutathione resin was added to pull-down GST and associated proteins, which were then separated by SDS-PAGE and stained with “InstantBlue.” “M” lanes have molecular weight markers. *Bottom*, loading controls—5% of the reaction volume was loaded on the gel. *Asterisks* mark the Yah1^GST^ degradation products (Fig. S3). *D*, quantification of three independent experiments carried out as described in *C*. The amounts of pulled down protein were quantified by densitometry and corrected for background binding to GST alone; protein levels in lane 3 (Yah1^GST^ interacting with NIA and Isu1_Sc_) were set at 100%. Error bars represent SD. *E*, Yah1^GST^ (2.5 μM) or GST (2.5 μM), as background control, was incubated with increasing concentrations of NIA in the absence (*gray line* and *circles*) or the presence (*black line* and *squares*) of Isu1_Ct_ (7.5 μM). Reaction mixtures were treated as described in *C*. The amounts of Nfs1 bound to Yah1^GST^, corrected for background binding to GST, from three independent experiments were plotted as relative units (RUs) with maximum binding of Nfs1 set to 1. Gels are shown in Fig. S4. *F*, BLI analysis of Yah1 interaction with NIA and NIA–Isu1. BLI sensors were loaded with Yah1^GST^ or GST (background control) as indicated. Association phase—at 30 s, loaded sensors were inserted into solutions containing NIA (10 μM) alone or together with Isu1 (10 μM), either Isu1_Sc_ or Isu1_Ct_. Dissociation phase—at 230 s, the sensors were placed in the solutions without proteins. *G*, BLI analysis of Yah1 interaction with Isu1. BLI sensors were loaded with Yah1^GST^ or GST (background control) as indicated. Association phase—at 30 s, loaded sensors were inserted into solutions containing Isu1_Sc_ (10 μM). Dissociation phase—at 230 s, the sensors were placed in the solutions without proteins. Representative experiments are shown; similar results were observed in three independent repeats. FeS, iron–sulfur; GST, glutathione-*S*-transferase; Isu1_Ct_, Isu1 ortholog of *C. thermophilum*; Isu1_Sc_, Isu1 ortholog of *S. cerevisiae*; NIA, Nfs1–Isd11–ACP; Yah1^GST^, Yah1-GST fusion protein.

Mitochondrial Nfs1 also forms a dimer, but it does not function alone. Instead, it forms a stable complex with a small accessory protein Isd11, which binds Nfs1 distal from the desulfurase active site. Isd11 is proposed to both stabilize Nfs1 and regulate its catalytic activity (28, 29, 30, 31, 32). In addition, recent structural analyses of the Nfs1–Isd11 complex revealed that Isd11 binds acyl carrier protein (ACP), thus the protomer of mitochondrial desulfurase consists of three proteins, forming the heterotrimer Nfs1–Isd11–ACP (9, 10, 33, 34). The role of ACP is unclear, as its absence does not affect *in vitro* desulfurase activity (9). However, *in vivo* analyses suggest that ACP has a regulatory function (35, 36). In this report, we refer to the dimer consisting of Nfs1–Isd11–ACP protomers as the NIA complex.

Structural data indicate that Nfs1 of NIA, when in complex with Isu1, adopts a conformation very similar to that of bacterial IscS (9). Furthermore, frataxin (Yfh1) interacts with the NIA–Isu1 complex in a manner similar to that reported for bacterial IscS binding in a cavity at the interface between Nfs1 and Isu1, interacting with both proteins (9, 10, 37). Neither Isd11 nor ACP are involved in the interactions between Yfh1 and the NIA–Isu1 complex. This binding mode is consistent with biochemical results indicating that Yfh1 interacts with the NIA complex only in the presence of prebound Isu1 (38, 39). However, to what extent the mode of ferredoxin (Yah1) binding to the NIA complex resembles those reported for its bacterial ortholog is unclear. Recent small-angle X-ray scattering analyses of biochemically reconstituted NIA complexes from human and thermophilic fungal (*Chaetomium thermophilum*) proteins point to simultaneous interaction of Yah1 and Yfh1 with the NIA–Isu1 complex (9). According to this model, Yfh1 interacts at its anticipated binding site, in a cavity between Nfs1 protomers and Isu1, whereas Yah1 binding is mediated by a novel interaction site, on the other side of Isu1. This mode of Yah1–Yfh1 interactions is very different from the mutually exclusive binding reported for bacterial IscS–IscU complex (27).

As the mode and timing of interaction of Yah1 and Yfh1 have potential mechanistic implications, we carried out biochemical experiments with purified WT and variant *S. cerevisiae* proteins to better understand the interaction of these components with NIA–Isu1. We found that interaction of Yah1 and Yfh1 with the NIA–Isu1 complex is mutually exclusive, as an excess of either Yfh1 or Yah1 displaces the other protein from a preformed complex with NIA–Isu1. Furthermore, we demonstrate that the same residues of Nfs1 are involved in the interaction with Yah1 and Yfh1. Consistent with these findings, unbiased molecular docking of Yah1 with a structural model of the NIA–Isu1 complex suggests overlapping binding sites for Yah1 and Yfh1. Taken together, we conclude that the Yah1 mode of interaction with the *S. cerevisiae* NIA–Isu1 complex resembles that observed for the bacterial ferredoxin interacting with the IscS–IscU complex and differs from the Yah1 binding mode reported for the human and *C. thermophilum* NIA–Isu1 complexes.

## Results

### Ferredoxin Yah1 binds NIA both in the absence and presence of Isu1

With a goal of analyzing the interaction of Yah1 with NIA and the effect of the presence of Isu1 (Fig. 1*A*), we used *Escherichia coli* expression systems to obtain purified components. To facilitate our studies, Yah1 was tagged at the C terminus with glutathione-*S*-transferase (GST). Holo-tagged (Yah1-GST fusion protein [Yah1^GST^]) and holo-untagged Yah1 were purified. To test whether the presence of the tag affected the properties of the cluster bound to Yah1, we measured the visible range of CD spectra of tagged and untagged versions (40). The spectra were indistinguishable, indicating that the presence of the tag does not affect the properties of the cluster (Fig. S1*A*). We also demonstrated that Yah1^GST^ is functional *in vivo*, as it supports the growth of yeast cells depleted of untagged Yah1 (Fig. S2). As described previously (39), the NIA complex was purified, *via* coexpression of Nfs1 and Isd11 under conditions that promote formation of an Nfs1–Isd11 heterodimer in complex with the endogenous *E. coli* ACP (ACP_Ec_) (9). We also purified the Isu ortholog of *C. thermophilum* (Isu1_Ct_), which can functionally replace *S. cerevisiae* Isu1 (Isu1_Sc_) both *in vivo* and *in vitro* (23, 41). Because of its enhanced stability, we used Isu1_Ct_ in experiments requiring high protein concentrations.

To detect interaction between Yah1 and NIA, we carried out pull-down assays using Yah1^GST^ as bait and glutathione beads to pull down Yah1^GST^ and any associated proteins (Fig. 1*B*). The presence of Nfs1, Isd11, and ACP_Ec_ in the pulled-down fraction indicated formation of a stable Yah1^GST^–NIA complex (Fig. 1, *C* and *D*, lane 2). To test whether the presence of Isu1 affects complex formation, we incubated Yah1^GST^ with a mixture of NIA and Isu1 (Fig. 1, *C* and *D*, lanes 3 and 5). All four proteins (Nfs1, Isd11, ACP_Ec_, and Isu1) were pulled down with Yah1^GST^, regardless of whether Isu1_Sc_ or Isu1_Ct_ was used. As no binding above the background level of either Isu1_Sc_ or Isu1_Ct_ to Yah1^GST^ was observed in the absence of NIA (Fig. 1, *C* and *D*, lanes 4 and 6), we concluded that a Yah1^GST^–NIA–Isu1 complex was formed. Reactions were then carried out with increasing concentrations of NIA. Concentration-dependent binding was observed both in the absence and presence of Isu1_Ct_ (Figs. 1*E* and S4). Furthermore, more binding was observed in the presence of Isu1_Ct_ at every concentration of NIA tested. These results indicate that NIA binds to Yah1^GST^ both in the absence and presence of Isu1. However, in the presence of Isu1, the Yah1^GST^–NIA complex is either more efficiently formed or more stable once formed.

The pull-down assay allows monitoring individual proteins interacting with Yah1^GST^. However, its disadvantage is that it provides a postequilibrium measurement. We therefore developed a biolayer interferometry (BLI) assay that allows time-resolved measurements of the Yah1–NIA interaction. Yah1^GST^ was immobilized on anti-GST coated BLI sensors, which were then introduced into solutions containing NIA alone, Isu1 alone, or a mixture of NIA and Isu1. NIA was rapidly recruited to the sensors carrying Yah1^GST^ (Fig. 1*F*) with equilibrium dissociation constant (*K*_*D*_) values in the micromolar range (Fig. S5), consistent with the results obtained using the pull-down assay. Because both binding assays described previously utilized the Yah1^GST^ fusion protein, we tested whether the presence of the GST tag affects the interaction of Yah1 with the NIA complex. To this end, we used a BLI assay in which His-tagged Nfs1 of the NIA complex was immobilized to a Ni–nitrilotriacetic acid-coated sensor, and interaction with untagged Yah1 was monitored (Fig. S6). We observed efficient Yah1 binding, which we interpret as indicating that the Yah1–NIA interaction is very similar regardless of whether the GST tag is present or not.

Furthermore, using the BLI assay, no direct interaction between Yah1^GST^ and Isu1_Sc_ was observed (Fig. 1*G*). However, in contrast to the pull-down results, the binding curves obtained for the NIA interaction with Yah1^GST^ (Fig. 1*F*) were very similar in the absence or presence of Isu1. One possible explanation of this apparent discrepancy between assays is that the presence of Isu1 does not affect the kinetics of the NIA binding but rather stabilizes the Yah1^GST^–NIA–Isu1 complex, which is particularly apparent under the harsh conditions of the pull-down experiment.

### Competition between ferredoxin Yah1 and frataxin Yfh1 for NIA–Isu1 binding

**Figure 2:**
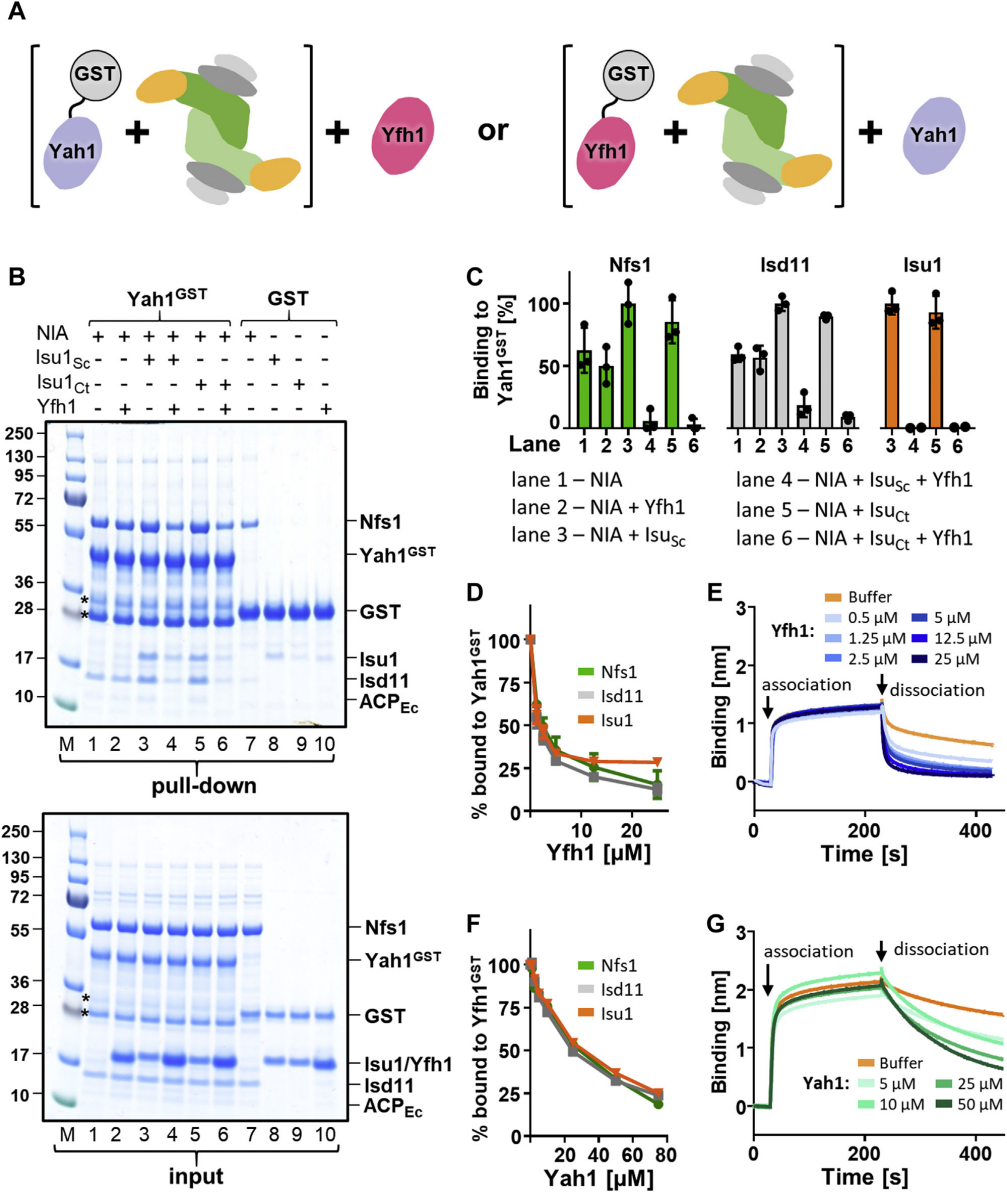
**Yah1 and Yfh1 compete for binding to the NIA–Isu1 complex**. *A*, general scheme of competition experiments. Either Yah1^GST^ (*left*) or Yfh1^GST^ (*right*) was incubated with NIA–Isu1 to allow complex formation; subsequently, an excess of either Yfh1 or Yah1 was added. *B*, *top*, Yah1^GST^ (2.5 μM) or GST (2.5 μM) was incubated with NIA (5 μM), Isu1_Sc_ (7.5 μM), Isu1_Ct_ (7.5 μM), and Yfh1 (12.5 μM), as indicated. Glutathione resin was added to pull-down GST and associated proteins, which were then separated by SDS-PAGE and stained with “InstantBlue.” *Bottom*, loading controls—5% of the reaction volume. *Asterisks* mark the Yah1^GST^ degradation products (Fig. S3). *C*, quantification of three independent experiments carried out as described in *B*. The amounts of pulled-down protein (Nfs1, Isd11, and Isu1) were quantified by densitometry and corrected for background binding to GST alone; protein levels in lane 3 (Yah1^GST^ interacting with NIA and Isu1_Sc_) were set as 100%. Error bars represent SD. *D*, Yfh1 displaces Yah1^GST^ from its complex with NIA–Isu1. Yah1^GST^ (2.5 μM) or GST (2.5 μM) (background control) was incubated with NIA (5 μM) and Isu1_Ct_ (7.5 μM) to allow complex formation. Then increasing concentrations of Yfh1 were added to the reaction mixtures. Reactions were treated as described in *B*. The quantities of Nfs1, Isd11, and Isu1_Ct_ pulled down with Yah1^GST^, corrected for background GST binding, were plotted for three independent experiments; the amount of protein bound to Yah1^GST^ in the absence of Yfh1 was set as 100%. Original gels are shown in Fig. S7*A*. *E*, BLI analysis of Yfh1 effect on the dissociation of the Yah1^GST^–NIA–Isu1 complex. Association phase—sensors loaded with Yah^GST^ were immersed at 30 s time point in solution containing NIA (10 μM) and Isu1_Ct_ (10 μM). Dissociation phase—at 230 s, the sensors were placed in solutions containing Yfh1 at indicated concentrations. A representative experiment is shown. Similar results were observed in three independent repeats. *F*, Yah1 competes with Yfh1^GST^ for binding to NIA–Isu1. Yfh1^GST^ (2.5 μM) or GST (2.5 μM) (background control) was incubated with NIA (5 μM) and Isu1_Ct_ (7.5 μM) to allow complex formation. Then increasing concentrations of Yah1 were added to the reaction mixtures. Reactions were treated as described in *B*. The quantities of Nfs1, Isd11, and Isu1_Ct_ pulled down with Yfh1^GST^, corrected for background GST binding, were plotted for three independent experiments; the amount of protein bound to Yfh1^GST^ in the absence of Yah1 was set as 100%. Original gels are shown in Fig. S7*B*. *G*, BLI analysis of the effect of Yah1 on dissociation of the Yfh1^GST^–NIA–Isu1 complex. Association phase—sensors loaded with Yfh^GST^ were immersed at 30 s time point into solution containing NIA (10 μM) and Isu1_Ct_ (10 μM). Dissociation phase—at 230 s, the sensors were placed into solutions containing Yah1 at indicating concentrations. A representative experiment is shown, and similar results were obtained in three independent repeats. BLI, biolayer interferometry; GST, glutathione-*S*-transferase; Isu1_Ct_, Isu1 ortholog of *C. thermophilum*; Isu1_Sc_, Isu1 ortholog of *S. cerevisiae*; NIA, Nfs1–Isd11–ACP; Yah1^GST^, Yah1-GST fusion protein; Yfh1^GST^, Yfh1-GST fusion protein.

We next asked whether ferredoxin Yah1 and frataxin Yfh1 can simultaneously interact with the NIA–Isu1 complex. We reasoned that if Yfh1 and Yah1^GST^ compete for the same binding site, excess Yfh1 should displace Yah1 from its complex with NIA–Isu1, thus reducing the amount of NIA–Isu1 complex pulled down with Yah1^GST^. Indeed, the amounts of Nfs1, Isd11, and Isu1 pulled down with Yah1^GST^ were reduced by 85% when a fivefold excess of Yfh1 was present in the reaction mixtures (Fig. 2, *B* and *C*, lanes 4 and 6), clearly indicating that Yfh1 displaced Yah1^GST^ from the NIA–Isu1 complex. When Isu1 was omitted, very little effect of the presence of Yfh1 on the pull down of NIA was observed (Fig. 2, *B* and *C*, lanes 1 and 2), consistent with previously published data showing that Yfh1 interaction with NIA strictly depends on the presence of Isu1 (39). Next, we carried out reactions with increasing concentrations of Yfh1. First, Yah1^GST^ was incubated with NIA and Isu1_Ct_ to preform the complex, and then increasing concentrations of Yfh1 were added to the reaction mixtures. Finally, glutathione resin was added to pull-down Yah1^GST^ and proteins bound to it. The amounts of Nfs1, Isd11, and Isu1_Ct_ pulled down with Yah1^GST^ decreased with increasing concentrations of Yfh1; at 5 μM Yfh1, the amounts were 30% of the control values measured in the absence of Yfh1 (Figs. 2*D* and S7). We conclude that Yfh1 is able to displace Yah1^GST^ from its complex with NIA–Isu1 in a concentration-dependent manner.

To test the effect of Yfh1 on the Yah1^GST^–NIA–Isu1 complex in a time-resolved manner, we used the BLI assay. BLI sensors with immobilized Yah1^GST^ were first immersed into a solution containing NIA and Isu1_Ct_ to allow Yah1^GST^–NIA–Isu1_Ct_ complex formation. Subsequently, sensors were immersed into solutions containing increasing concentrations of Yfh1. The complex disassembled faster in the presence of increasing concentrations of Yfh1, as indicated by steeper dissociation curves (Fig. 2*E*) and the increasing values of dissociation rate (*k*_*d*_) constants in the presence of Yfh1 (Fig. S8). We interpret this result as indicating that Yfh1, *via* binding to the NIA–Isu1 released from the complex with Yah1^GST^, prevents its rebinding to Yah1^GST^, thus increasing the observed *k*_*d*_.

To test whether Yah1 can displace Yfh1 from its complex with NIA–Isu1, we took advantage of our previous work (39) showing that the Yfh1-GST fusion protein (Yfh1^GST^), which like Yah1^GST^ is functional *in vivo* (42), can be used to study the Yfh1–NIA–Isu1 interaction. Indeed, when the preformed Yfh1^GST^–NIA–Isu1 complex was incubated with increasing concentrations of Yah1, we observed reduced amounts of Nfs1, Isd11, and Isu1_Ct_ pulled down with Yfh1^GST^ (Figs. 2*F* and S7). However, Yah1 was a less efficient competitor than Yfh1, as a 75 μM concentration of Yah1 was needed to reduce the amounts of Nfs1, Isd11, and Isu1_Ct_ pulled down with Yfh1^GST^ to 35% of the control value measured in the absence of Yah1. This result was further verified using the BLI assay (Fig. 2*G*). First, we assembled the Yfh1^GST^–NIA–Isu1 complexes by inserting sensors with immobilized Yfh1^GST^ into solutions containing NIA and Isu1_Ct_. Next, these sensors were immersed in solutions containing increasing concentrations of Yah1. The steeper dissociation curves as well as the increased values of the *k*_*d*_ constants in the presence of Yah1 (Fig. S9) indicate that it prevents rebinding of the NIA–Isu1 released from the complex with Yfh1^GST^, although high concentrations are required. Taken together, these competition experiments clearly demonstrated that binding of ferredoxin Yah1 and frataxin Yfh1 to the NIA–Isu1 complex is mutually exclusive. Moreover, low concentrations of Yfh1 were sufficient to displace Yah1^GST^ from the Yah1^GST^–NIA–Isu1 complex, indicating that under conditions used in these experiments, Yfh1 has higher affinity for NIA–Isu1 than Yah1 does.

### Residues involved in the interaction of Yah1 with NIA

**Figure 3:**
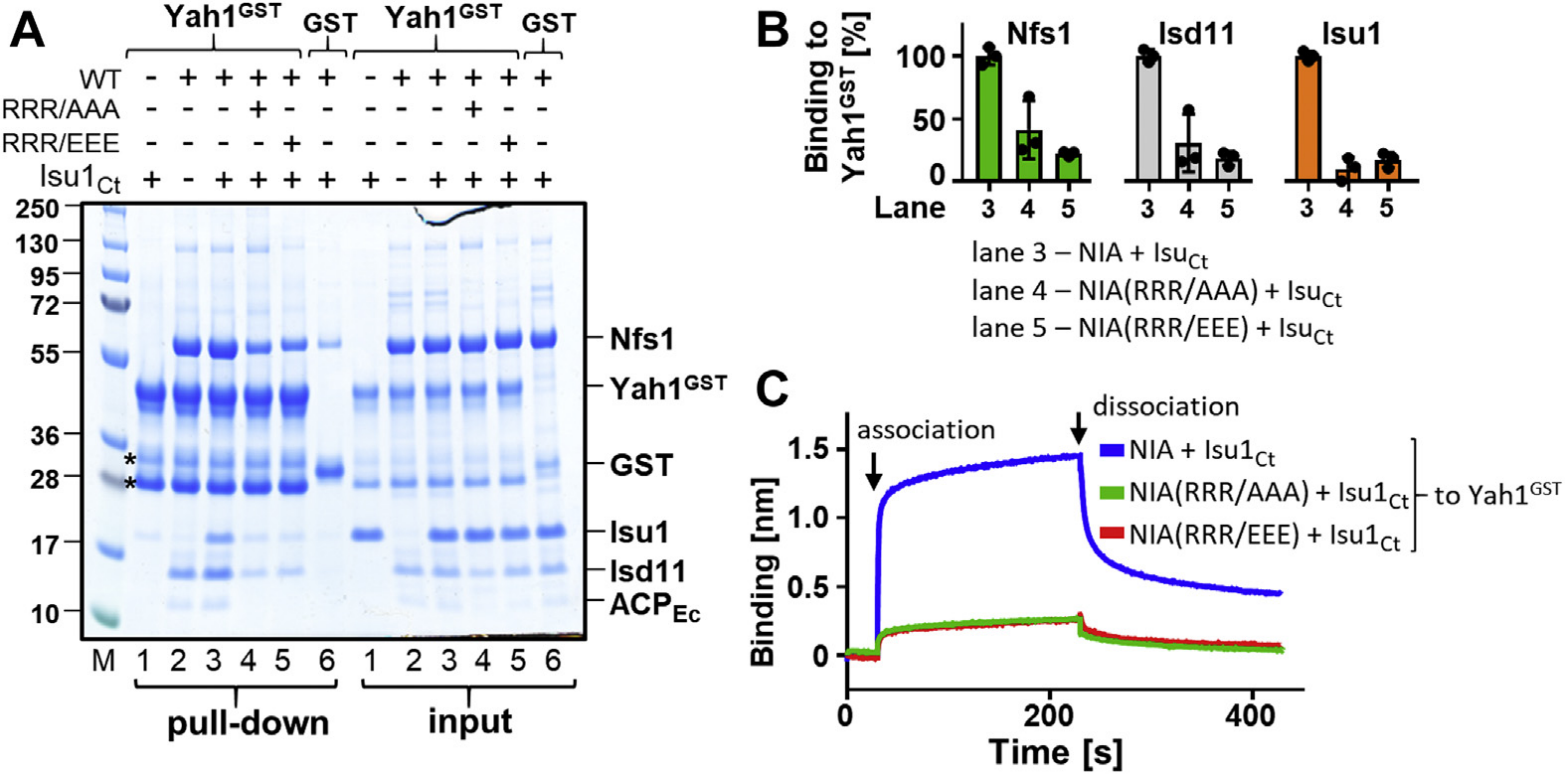
**Evolutionary conserved residues of Nfs1 (Arg**^**313**^**, Arg**^**316**^**, and Arg**^**318**^**) are critical for Yah1 interaction with NIA–Isu1 complex.***A*, Yah1^GST^ (2.5 μM) or GST (2.5 μM) was incubated with Isu1_Ct_ (7.5 μM) and NIA (5 μM) containing either Nfs1 WT or Nfs1 substitution variants: Nfs1(R^313^A, R^316^A, and R^318^A) (RRR/AAA); Nfs1(R^313^E, R^316^E, and R^318^E) (RRR/EEE). Glutathione resin was added to pull-down GST and associated proteins, which were then separated by SDS-PAGE and stained with “InstantBlue.” Input-loading control; 5% of the reaction volume was loaded on the gel. “M” indicates lane having molecular weight marker. *Asterisks* mark the Yah1^GST^ degradation products (Fig. S3). *B*, quantification of three independent experiments carried out as described in *A*. The quantities of Nfs1, Isd11, and Isu1_Ct_ pulled down with Yah1^GST^ and corrected for background GST binding were plotted for three independent experiments; the protein levels bound to NIA containing WT Nfs1 (lane 3) were set at 100%. *C*, BLI analysis of Nfs1 (RRR/AAA) and (RRR/EEE) variants binding to Yah1^GST^. Association phase—sensors loaded with Yah^GST^ were immersed at 30 s time point in solution containing Isu1_Ct_ (10 μM) and NIA (10 μM) and either Nfs1 WT or variants (RRR/AAA) and (RRR/EEE) as indicated. Dissociation phase—at 230 s, the sensors were placed in solutions without proteins. A representative experiment is shown. Similar result was observed in three independent repeats. BLI, biolayer interferometry; GST, glutathione-*S*-transferase; Isu1_Ct_, Isu1 ortholog of *C. thermophilum*; NIA, Nfs1–Isd11–ACP; Yah1^GST^, Yah1-GST fusion protein.

A simple interpretation of the mutually exclusive binding of Yah1 and Yfh1 to the NIA–Isu1 complex is that these proteins utilize an overlapping binding site. We demonstrated previously that evolutionarily conserved arginine residues (Arg^313^, Arg^316^, and Arg^318^) of Nfs1 are critical for Yfh1 binding to the NIA–Isu1 complex (39). The physiological importance of these residues is supported by the null growth phenotype of yeast strains carrying Nfs1 variants having substitutions of these residues (39). To test whether these residues are also important for Yah1 binding, we purified NIA complexes containing two Nfs1 variants having substitutions of these residues—either to alanine (R313A, R316A, and R318A) or to a residue with an opposite charge (R313E, R316E, and R318E). We then measured, using both pull-down and BLI assays, their ability to bind Yah1^GST^. In the pull-down assay, we observed that binding of NIA–Isu1 complexes containing Nfs1 variants to Yah1^GST^ was greatly reduced, with Nfs1(RRR/AAA) and Nfs1(RRR/EEE) variants binding at 30% and 20% of the level of WT control, respectively (Fig. 3, *A* and *B*, lanes 3–5). Similar results were obtained using the BLI assay (Figs. 3*C* and S10). Both NIA–Isu1 complexes containing Nfs1(RRR/AAA) and Nfs1(RRR/EEE) variants interacted very weakly with immobilized Yah1^GST^, in comparison to the WT control. These results clearly demonstrated that the arginine residues of Nfs1 that are required for the Yfh1–NIA–Isu1 interaction are also critical for Yah1 binding.

**Figure 4:**
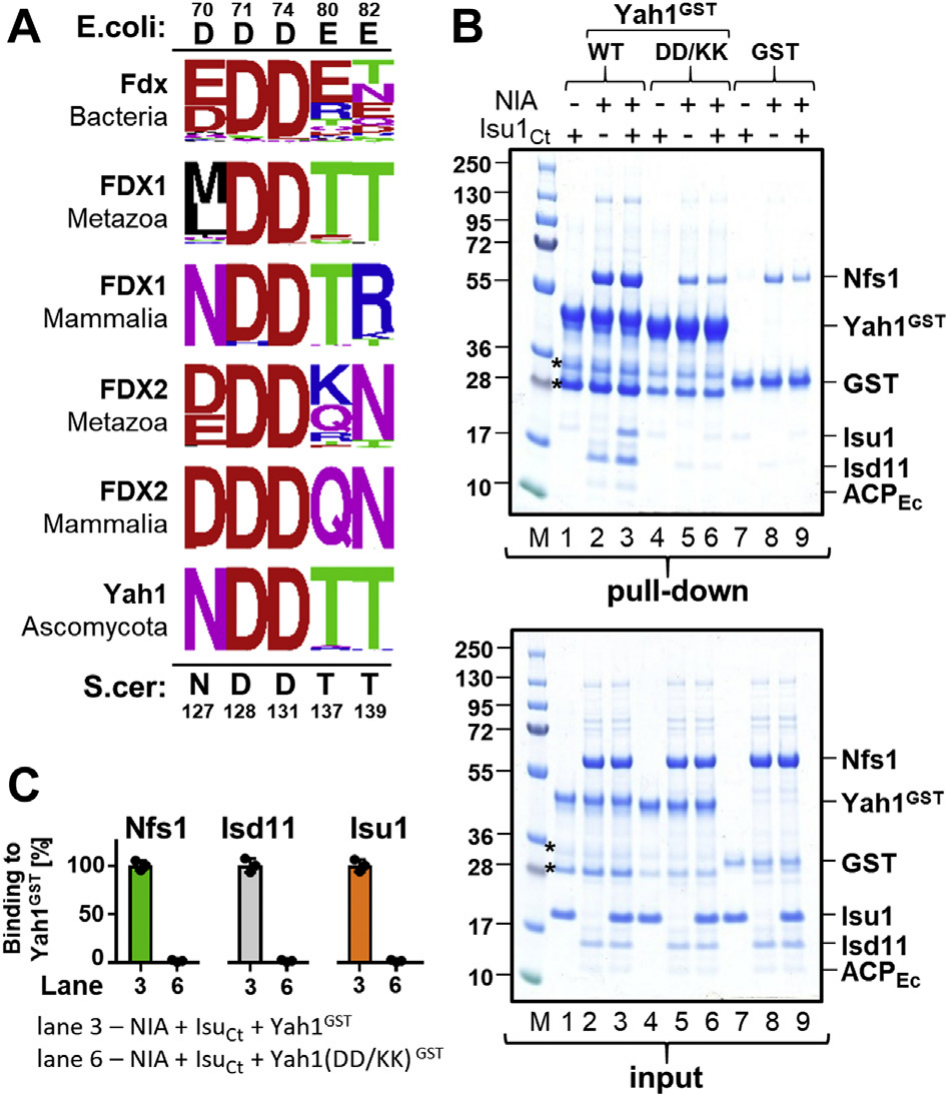
**Evolutionary conserved residues of Yah1 (Asp**^**128**^**and Asp**^**131**^**) are critical for the NIA–Isu1 binding.***A*, sequence conservation at positions, which in *Escherichia coli* Fdx, is involved in the interaction with IscS (27). Frequency logos are shown for ferredoxin orthologs from Proteobacteria, Metazoa—excluding mammals, Mammalia, and Ascomycota. *E. coli* Fdx residues interacting with IscS and homologous *Saccharomyces cerevisiae* Yah1 residues are shown at *top* and *bottom*, respectively. Note that only Asp^71^ and Asp^74^ of *E. coli* Fdx are conserved across phylogeny, including *S. cerevisiae* Yah1 residues Asp^128^ and Asp^131^. *B*, *top*, Yah1^GST^ (WT), Yah1 (D^128^K and D^131^K)^GST^ (DD/KK), or GST (2.5 μM) was incubated with NIA (5 μM) and Isu1_Ct_ (7.5 μM), as indicated. Glutathione resin was added to pull-down GST and associated proteins, which were then separated by SDS-PAGE and stained with “InstantBlue.” *Bottom*, loading controls—5% of the reaction volume was loaded on the gel. *Asterisks* mark the Yah1^GST^ degradation products (Fig. S3). *C*, quantification of three independent experiments carried out as described in *B*. The quantities of Nfs1, Isd11, Isu1_Ct_ pulled down with Yah1^GST^ WT or (D^128^K and D^131^K) variant (DD/KK), corrected for background GST binding, were plotted; the protein levels bound to Yah1^GST^ WT (lane 3) were set as 100%. Error bars represent SD. Isu1_Ct_, Isu1 ortholog of *C. thermophilum*; NIA, Nfs1–Isd11–ACP; Yah1^GST^, Yah1-GST fusion protein.

The dependence of the Yah1–Nfs1 interaction on conserved Nfs1 arginine residues suggested to us that Yah1 residues involved in this interaction may also be evolutionarily conserved. Therefore, we analyzed the evolutionary conservation of the five residues of *E. coli* ferredoxin Fdx previously shown to be involved in its interaction with cysteine desulfurase IscS—Asp^70^, Asp^71^, Asp^74^, Glu^80^, and Glu^82^ (27). To this end, we assembled a dataset of 473 ferredoxin orthologs from 208 bacterial, 69 fungal, 86 metazoan, including 22 mammalian species. These sequences were aligned, and protein phylogeny was inferred using the maximum likelihood method (Fig. S11). Only Asp^71^ and Asp^74^ of *E. coli* Fdx, corresponding to Asp^128^ and Asp^131^ of *S. cerevisiae* Yah1, are universally conserved among ferredoxin orthologs (Fig. 4*A*). Charge reversal substitutions do not support growth of cells having WT Yah1 expression repressed (Fig. S2*B*).

Because two paralogous ferredoxins (FDX1 and FDX2) coexist in metazoans, including mammals, we used protein phylogeny to identify FDX1 and FDX2 orthologs in our metazoan dataset. Those that clustered with FDX1 or FDX2 were assigned as their respective orthologs. Evidence indicates that in mammalian mitochondria, FDX2 functions in the biogenesis of FeS clusters (9, 19, 20, 23, 43), whereas the role of FDX1 in this process is under debate (21). However, these aspartate residues are conserved in both FDX1 and FDX2 paralogs.

To test experimentally whether these conserved residues are indeed important for Yah1–NIA complex formation, we purified untagged and GST-tagged versions of a Yah1 variant having Asp^128^ and Asp^131^ residues replaced by lysine, Yah1(DD/KK). Before initiating biochemical analyses, we measured the UV range CD spectra and *T*_M_ of purified Yah1(DD/KK) variant to verify whether the substitutions affected overall structure and stability; no significant differences were detected in comparison to the WT control (Fig. S12, *A* and *B*). Furthermore, the visible range CD spectra of Yah1(DD/KK) were indistinguishable from the WT control, indicating that the substitutions did not affect the properties of the FeS cluster bound to Yah1 (Fig. S12*C*). When Yah1(DD/KK)^GST^ was tested in the pull-down assay, the amounts of Nfs1 and Isd11 bound to it were reduced to less than 5% of the WT control value, both in the absence or presence of Isu1_Ct_ (Fig. 4, *B* and *C*).

**Figure 5:**
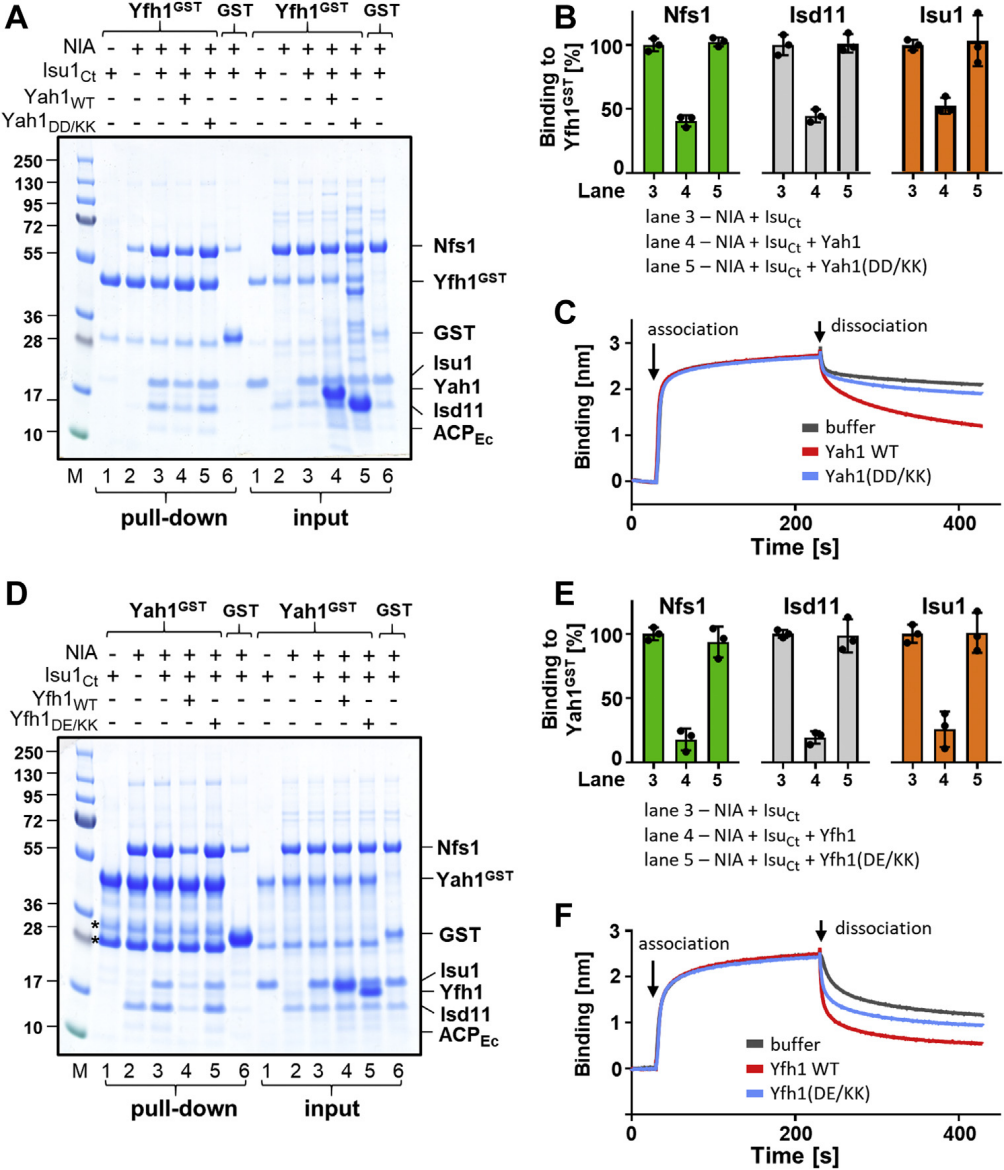
**Yah1 and Yfh1 variants defective in Nfs1 interaction are unable to displace each other from the NIA–Isu1 complex.***A*, Yfh1^GST^ or GST (at 2.5 μM) was incubated with NIA (5 μM), Isu1_Ct_ (7.5 μM), and Yah1 (WT) or Yah1(D^128^K, D^131^K) (DD/KK) (50 μM). Glutathione resin was added to pull-down GST and associated proteins, which were then separated by SDS-PAGE and stained with “InstantBlue.” Input-loading control of 5% of the reaction volume. *Asterisks* mark the Yah1^GST^ degradation products (Fig. S3). *B*, quantification of three independent experiments carried out as described in *A*. Bound proteins (Nfs1, Isd11, and Isu1) were quantitated by densitometry and corrected for background binding to GST alone; the amount of protein bound to Yfh1^GST^ in the presence of Isu1_Ct_ (lane 3) was set as 100%. Error bars represent SD. *C*, BLI analysis of Yah1(DD/KK) effect on dissociation of the preformed Yfh1^GST^–NIA–Isu1 complex. Association phase—sensors loaded with Yfh1^GST^ were immersed at 30 s time point into solution containing NIA and Isu1_Ct_ mixture (at 10 μM). Dissociation phase—at 230 s, the sensors were placed in solutions containing Yah1 (WT) or Yah1(DD/KK) (at 40 μM) or solution without proteins (buffer). Representative experiment is shown, and similar results were observed in three independent repeats. *D*, Yah1^GST^ or GST (at 2.5 μM) was incubated with NIA (5 μM), Isu1_Ct_ (7.5 μM), and Yfh1 (WT) or Yfh1(D^86^K, E^89^K) (DE/KK) (at 12.5 μM), as indicated. The samples were treated as in *A*. *E*, quantification of three independent experiments carried out as described in *D*. Bound proteins were quantitated as in *B*. The amount of protein bound to Yah1^GST^ in the presence of Isu1_Ct_ (lane 3) was set as 100%; error bars represent SD. *F*, BLI analysis of Yfh1(DE/KK) effect on dissociation of the preformed Yah1^GST^–NIA–Isu1 complex. Association phase—sensors loaded with Yah1^GST^ were immersed at 30 s time point into solution containing NIA and Isu1_Ct_ mixture (at 10 μM). Dissociation phase—at 230 s, the sensors were placed in solutions containing Yfh1 (WT) or Yfh1(DE/KK) (at 12.5 μM) or solution without proteins (buffer). Representative experiment is shown, and similar results were observed in three independent repeats. BLI, biolayer interferometry; GST, glutathione-*S*-transferase; Isu1_Ct_, Isu1 ortholog of *C. thermophilum*; NIA, Nfs1–Isd11–ACP; Yah1^GST^, Yah1-GST fusion protein; Yfh1^GST^, Yfh1-GST fusion protein.

Based on these results, we also hypothesized that Asp^128^ and Asp^131^ of Yah1 are most likely interacting with Arg^313^, Arg^316^, and Arg^318^ of Nfs1, suggesting that Yah1 and Yfh1 use the same set of evolutionary conserved Nfs1 residues for their interaction with NIA–Isu1. If this is indeed the case, we reasoned that neither Yah1 nor Yfh1 variants defective in Nfs1 interaction would be able to displace the other protein from its complex with NIA–Isu1. To verify this prediction, we not only repeated the type of competition experiments described previously but also included Yah1 and Yfh1 variants defective in their interaction with Nfs1. When preformed Yfh1^GST^–NIA–Isu1 was incubated with excess Yah1(DD/KK), the amounts of Nfs1, Isd11, and Isu1 pulled down were the same as in the control lacking Yah1 (Fig. 5, *A* and *B*), indicating that Yah1(DD/KK), which is defective in Nfs1 binding, was not able to compete with Yfh1 for the NIA–Isu1 interaction. Complementary results were obtained when preformed Yah1^GST^–NIA–Isu1 complex was incubated with excess Yfh1(D86K, E89K) variant, which we have shown previously to be defective in the Nfs1 interaction *in vitro* and to cause a slow growth phenotype *in vivo* (39). Yfh1(DE/KK) was not able to displace Yah1^GST^ from its complex with NIA–Isu1 (Fig. 5, *D* and *E*). Finally, the BLI measurements revealed that neither Yah1 variants nor Yfh1 variants defective in the Nfs1 interaction were able to increase the observed *k*_*d*_s of preassembled Yfh1^GST^ and Yah1^GST^ complexes with NIA–Isu1 to the extent observed for the WT proteins (Figs. 5, *C* and *F*, S13, and S14). Taken together, the results of these competition experiments further support the notion that the binding site of Yah1 overlaps with the binding site of Yfh1 and that their interactions with Nfs1 involve the same set of evolutionary conserved arginine residues. However, we cannot exclude the possibility that Yfh1 binding changes the conformation of NIA in a way that decreases the binding affinity for Yah1.

### Yah1 residues involved in the Nfs1 interaction are critical for the NIA–Isu1–Yah1 complex formation under anaerobic conditions

**Figure 6:**
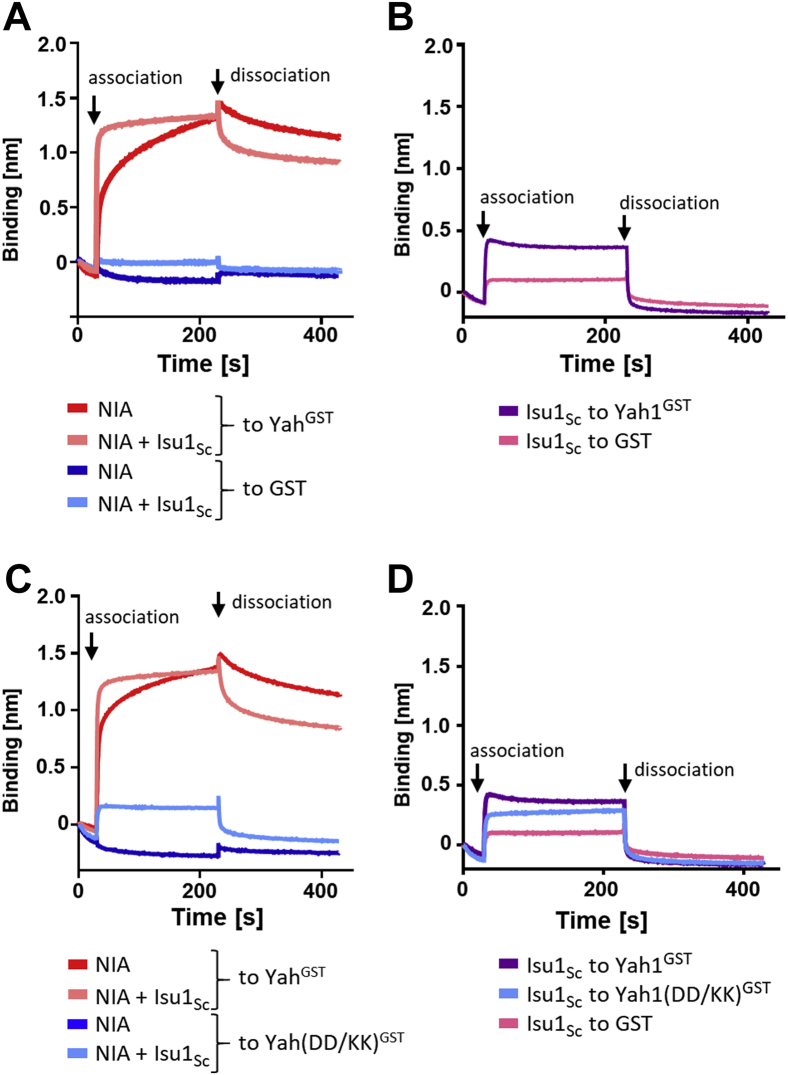
**Conserved Asp**^**128**^**and Asp**^**131**^**are critical for interaction of chemically reduced Yah1**^**GST**^**with NIA–Isu1 under anaerobic conditions.***A* and *B*, anaerobic BLI analysis of chemically reduced Yah1^GST^ interaction with NIA and NIA–Isu1 (*A*) or Isu1 (*B*). BLI sensors were loaded with Yah1^GST^ or GST (background control) as indicated. Association phase—at 30 s, loaded sensors were inserted into solutions containing NIA (10 μM) alone or together with Isu1_Sc_ (10 μM) (*A*) or Isu1_Sc_ (10 μM) (*B*). Dissociation phase—at 230 s, the sensors were placed in the solutions without proteins. *C* and *D*, anaerobic BLI analysis of NIA, NIA–Isu1, and Isu1 binding to chemically reduced Yah1^GST^(DD/KK) variant. Association phase—sensors loaded with Yah1^GST^ WT, DD/KK, or GST alone, as indicated, were immersed at 30 s time point into solution containing NIA (10 μM), NIA and Isu1_Sc_ mixture (at 10 μM) (*C*), and Isu1_Sc_ (10 μM) (*D*). Dissociation phase—at 230 s, the sensors were placed in solutions without proteins. Representative experiments are shown; similar results were obtained in three independent repeats. BLI, biolayer interferometry; GST, glutathione-*S*-transferase; Isu1_Sc_, Isu1 ortholog of *S. cerevisiae*; NIA, Nfs1–Isd11–ACP; Yah1^GST^, Yah1-GST fusion protein.

The experiments described previously were performed under aerobic conditions, thus the [2Fe–2S] cluster bound to Yah1 was in the oxidized form. However, during FeS cluster biosynthesis, Yah1 functions in the reduced form. Furthermore, evidence indicates that in the reduced form, Yah1 directly interacts with Isu1 (23) and that this interaction plays a role in its binding to human and *C. thermophilum* NIA–Isu1 complexes (9). To test whether reduction of Yah1 affects its interaction with NIA, we carried out BLI assays under anaerobic conditions using chemically reduced Yah1^GST^ (Fig. 6*A*). To assess whether the GST tag affects the properties of cluster bound to Yah1^GST^, we measured the visible range CD spectra (40); the spectra were indistinguishable from the WT control (Fig. S1*B*). The binding curve obtained for NIA interaction with reduced Yah1^GST^ in the presence of Isu1_Sc_ was similar to that observed under aerobic conditions, that is, when the Yah1 [2Fe–2S] cluster is in the oxidized form (Fig. 1*F*). However, the maximum binding signal was 30% higher and the complex more stable, as indicated by a very shallow dissociation curve (Figs. 6*A* and S15). We also observed slower binding of NIA in the absence of Isu1_Sc_, whereas under aerobic conditions, the two curves were very similar (Fig. 1*F*). Despite these differences between reduced and oxidized Yah1, the results of our analyses indicate that under both anaerobic and aerobic conditions, Yah1 binds NIA very efficiently either in the absence or the presence of Isu1.

We also detected direct interaction between reduced Yah1^GST^ and Isu1_Sc_ (Fig. 6*B*), although as discussed previously, we did not observe such interaction under aerobic conditions either by BLI or pull-down assays (Fig. 1, *C* and *G*). To test whether the direct Yah1–Isu1 interaction might be sufficient for Yah1–NIA–Isu1 complex formation under anaerobic conditions, we used the Yah1(DD/KK) variant defective in interaction with Nfs1. Chemically reduced Yah1(DD/KK)^GST^ maintained the ability to interact with Isu1; the Isu1_Sc_ binding signal was reduced by only 10% in comparison to WT Yah1^GST^ in the BLI assay performed under anaerobic conditions (Fig. 6*D*). However, a dramatic reduction in NIA and NIA–Isu1 binding was observed with chemically reduced Yah1(DD/KK)^GST^ variant immobilized to the BLI sensor (Figs. 6*C* and S16). Taken together, these results indicate that Asp^128^ and Asp^131^ residues are critical for Yah1^GST^ binding to both NIA and NIA–Isu1 complexes under anaerobic conditions, and that the Yah1–Isu1 interaction, which is maintained in the Yah1(DD/KK)^GST^ variant, is not sufficient for Yah1^GST^–NIA–Isu1 complex formation.

### Structural model of Yah1 interacting with NIA–Isu1 complex

**Figure 7:**
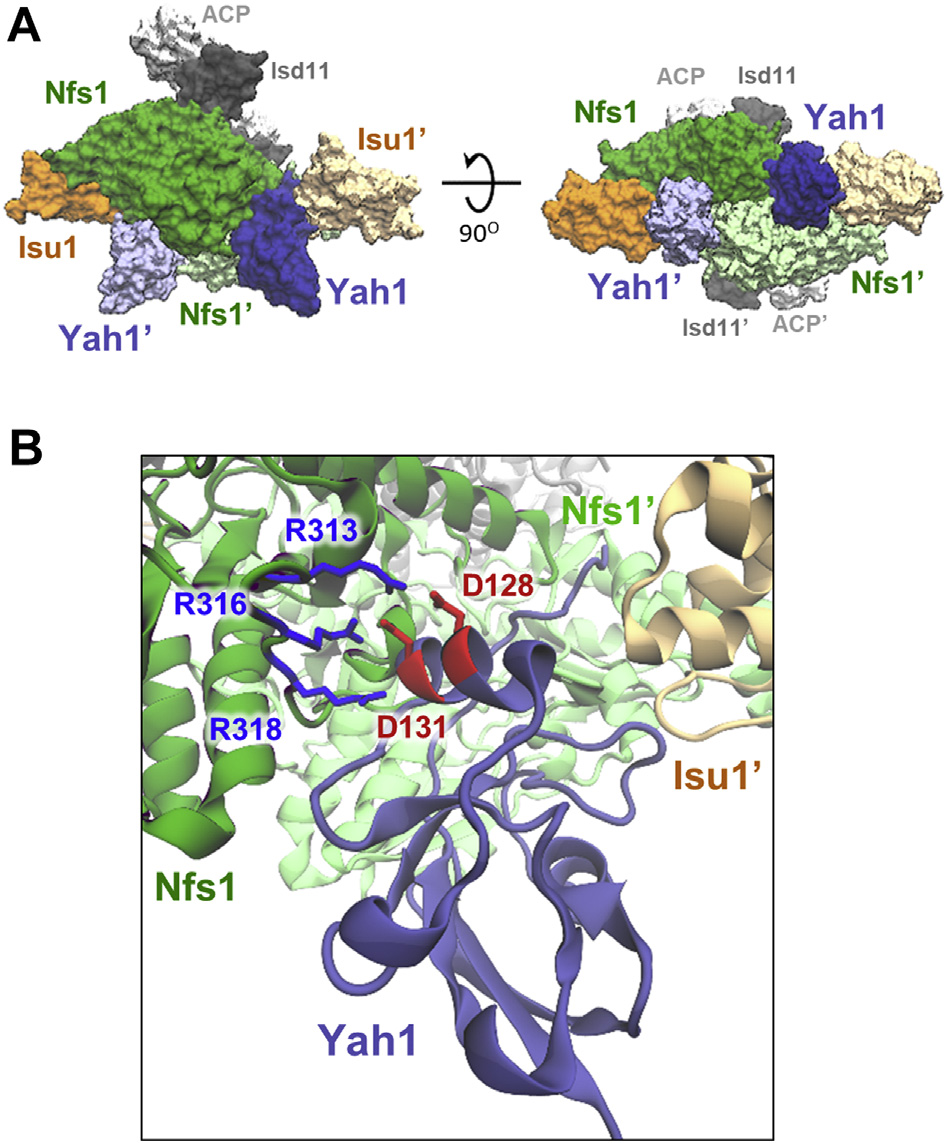
**Structural model of Yah1 interacting with NIA–Isu1 complex.***A*, unbiased molecular docking of Yah1 structure (Protein Data Bank: 2MJD) to homology model of the NIA–Isu1 complex was used to obtain this structural model. Yah1 (*blue*) and Yah1' (*light blue*) interact with the NIA–Isu1 complex in a cleft between Nfs1 (*green*) and Nfs1' (*light green*) protomers and Isu1 (*orange*) and Isu1' (*light orange*) bound to them. Isd11 (*dark gray*) and ACP (*light gray*) do not participate in these interactions. The mode of Yah1 binding resembles bacterial Fdx interacting with IscS dimer. *B*, zoom in on the binding interface. Asp^128^ and Asp^131^ of Yah1 are in contact with Arg^313^, Arg^316^, and Arg^318^ of Nfs1. ACP, acyl carrier protein; NIA, Nfs1–Isd11–ACP.

Using biochemical assays, we identified residues important for Yah1 interaction with the NIA–Isu1 complex. To better inform the mode of this interaction, we used unbiased molecular docking to obtain a structural model of Yah1 interaction with the NIA–Isu1 complex. To this end, we used the Yah1 structure (Protein Data Bank [PDB]: 2MJD) and a model of the NIA–Isu1 complex obtained *via* superimposition of homology models of *S. cerevisiae* Nfs1, Isd11, and Isu1 proteins on a cryo-EM structure of the human NIA–Isu1 complex (PDB: 6NZU) (Fig. 7*A*). Yah1 docking using the ClusPro webserver predicted a binding mode (six of 10 top scoring structures), in which an ensemble of Yah1 structures interacts in a cleft between Nfs1 protomers next to the Isu1 bound to Nfs1—the location of the Yfh1 binding site (Fig. 7*A*). This binding mode is consistent with the results of our biochemical experiments as it predicts contacts between aspartate residues of Yah1 (Asp^128^ and Asp^131^) and arginine residues of Nfs1 (Arg^313^, Arg^316^, and Arg^318^) (Fig. 7*B*). Taken together, the computationally predicted mode of Yah1–NIA–Isu1 interaction is consistent with our biochemical results and very similar to the mode of bacterial Fdx–IscS–IscU interaction (27).

## Discussion

Several lines of evidence presented here indicate that in the *S. cerevisiae*, interaction of ferredoxin (Yah1) and frataxin (Yfh1) with the NIA–Isu1 complex is mediated by overlapping binding sites on Nfs1 and is thus mutually exclusive: (i) no simultaneous binding of Yah1 and Yfh1 to NIA–Isu1 was detected using biochemical assays; instead, each was able to displace the other from its complex with NIA–Isu1, (ii) the same evolutionarily conserved Nfs1 arginine residues were critical for interaction of both Yah1 and Yfh1 with the NIA–Isu1 complex, (iii) when Yah1 residues critical for Nfs1 binding were substituted, its ability to interact with NIA was aggravated; this variant did not displace Yfh1 from the complex with NIA–Isu1, and (iv) unbiased molecular docking of Yah1 to the NIA–Isu1 complex predicted that Yah1 interacts with Nfs1 at a site overlapping with the Yfh1 binding site.

Despite the mutually exclusive binding mode and reliance on at least some of the same residues of Nfs1, the interactions of ferredoxin Yah1 and frataxin Yfh1 with NIA complex differ significantly. It is well established that frataxin binding requires the presence of Isu1; such dependence has been reported for bacterial (26), mammalian (38), and fungal (39) systems. Moreover, biochemical and structural analyses have revealed that frataxin interacts with both Nfs1 and Isu1 when in complex with NIA–Isu1. However, we did not observe a critical dependence on Isu1 for the interaction of Yah1 with NIA, as Yah1 binding was efficient even in its absence—consistent with the direct interaction between ferredoxin and cysteine desulfurase reported for bacterial orthologs (22, 27). However, Yah1 binding is slightly more efficient in the presence of Isu1. This difference could be explained by the direct interaction of Yah1 with Isu1, which was also reported previously for *S. cerevisiae* (23) as well as for human and *C. thermophilum* orthologs (9). Though both interactions could be contributing to complex stability, the contributions are highly skewed. The Yah1–Nfs1 interaction is critical for complex formation—as evidenced by the inability of a Yah1 mutant (Yah1(DD/KK)) defective in the interaction with Nfs1 but able to interact with Isu1 to form a detectable Yah1–NIA–Isu1 complex. Alternatively, more efficient Yah1 binding to the NIA–Isu1 complex could be explained by the higher stability of such a complex in comparison to the one with NIA alone, as conformational changes of the Nfs1 dimer interface were observed upon Isu1 interaction with NIA (9).

Our results demonstrate that while both Yah1 and Yfh1 are competing for binding to NIA–Isu1, Yfh1 is a more potent competitor. Low concentrations are sufficient to displace Yah1 from its complex with NIA–Isu1, whereas high concentrations of Yah1 are required for the inverse reaction. Are these differences consistent with the predicted functions of Yfh1 and Yah1 during the FeS cluster biosynthesis? According to recently published results (19, 44), Yfh1 functions in FeS cluster biosynthesis before Yah1: Yfh1 accelerates persulfide transfer from the cysteine desulfurase onto iron-loaded Isu1 bound to NIA; once persulfide is loaded onto Isu1, it is reduced to sulfide by Yah1. Thus, it makes sense that Yfh1 has a high affinity for the initial NIA–Isu1 complex. But, then how does Yah1 gets access to the Isu1-bound persulfide? Perhaps, Isu1 changes conformation upon persulfide addition such that either its interaction with Yfh1 is weakened or that with Yah1 is strengthened, making Yah1 able to compete it out of the NIA–Isu1 complex. Since our competition assays did not use persulfide-loaded Isu1, further mechanistic studies are needed to verify this scenario.

Overall, our results revealed that *S. cerevisiae* ferredoxin (Yah1) and frataxin (Yfh1) interactions with NIA–Isu1 complex are mutually exclusive and thus similar to the binding mode reported previously for bacterial ferredoxin (Fdx) and frataxin (CyaY) interacting with cysteine desulfurase (IscS) (27). Thus, supporting a view that FeS cluster assembly machinery functioning in *S. cerevisiae* was inherited from bacterial ancestors of mitochondria and that the presence of accessory proteins (*i.e.*, Isd11 and ACP) has not changed the mode of frataxin–ferredoxin interaction with Nfs1. However, our findings differ significantly from the ferredoxin-binding mode reported recently for biochemically reconstituted human and thermophilic fungal (*C. thermophilum*) NIA–Isu1 complexes (9). Structural models of these complexes, based on small-angle X-ray scattering, predict simultaneous binding of ferredoxin and frataxin to the NIA–Isu1 complex. In these models, frataxin interacts with the NIA–Isu1 complex at its typical binding site in a cleft between Nfs1 dimerization interface and Isu1 bound to Nfs1. Thus, the frataxin-binding mode is conserved among all NIA–Isu1 complexes tested so far (9, 10), including the one presented in this report. However, the binding mode of ferredoxin differs significantly, as it was proposed that human and *C. thermophilum* ferredoxin interacts with the NIA–Isu1 complex at a novel binding site. This site does not overlap with the frataxin interaction interface, as it is on the opposite side of Isu1.

Such contrasting ferredoxin-binding modes between different fungi and metazoan lineages are surprising and difficult to reconcile with their evolutionary history, considering that fungi and metazoans belong to sister clades sharing common ancestry (45). Furthermore, our evolutionary analysis has revealed that the aspartate residues that are critical for the *S. cerevisiae* Yah1 interaction with NIA–Isu1 complex at the binding site it shares with frataxin are conserved across ferredoxin orthologs from bacterial, fungi, and metazoan species, including mammals. Clearly, further biochemical, biophysical, and evolutionary studies are needed to explain how a new mode of ferredoxin interaction has evolved and how it is functionally related to the universally conserved frataxin-binding interface.

## Experimental procedures

### Protein purification

Recombinant WT and substitution variants of *S. cerevisiae* Yah1 were purified as described (23) with some modifications. BL21(DE3) cells transformed with pETDuet1Yah1_Sc_ were grown in LB at 30 °C, induced with 1 mM IPTG at an absorbance of 0.6 at 600 nm, and supplemented with 50 μM ferric ammonium citrate during the overnight induction period. Cells were harvested by centrifugation, frozen, thawed, and lysed by French press in Q1 buffer (50 mM Tris–HCl, pH 7.5; 10% [v/v] glycerol; 50 mM NaCl; 2 mM EDTA, and pH 7.5) containing 1 mM PMSF to inhibit proteolysis. The soluble brown supernatant fluid following centrifugation at 75,000*g* was used for purification, and all subsequent chromatography studies were carried out in Q1 buffer at 4 °C. Cell lysates containing either WT Yah1 or its substitution variants were initially purified using Q-sepharose. Soluble extracts were applied to the column and washed with several column volumes of Q1 buffer containing 1 mM PMSF. Yah1 was eluted using a linear gradient of 100 mM to 500 mM NaCl in buffer Q1, and fractions were analyzed by gel electrophoresis, then combined, and concentrated using Amicon Ultra-15 Centrifugal Filter Units and dialyzed against P1 buffer (50 mM Tris–HCl [pH 7.5]; 50 mM NaCl; 2 mM EDTA [pH 7.5]). After dialysis, a sample of Yah1 was supplemented with ammonium sulfate to 2 M concentration and applied to a Phenyl Sepharose column. Yah1 was eluted with a linear gradient decreasing from 2 M to zero ammonium sulfate. Fractions containing Yah1 were concentrated by ultrafiltration and subjected to buffer exchange to buffer F (50 mM Tris–HCl [pH 7.5]; 10% glycerol; 50 mM NaCl) during the dialysis step. The final preparation of Yah1 was aliquoted, frozen in liquid nitrogen, and stored at −70 °C.

Expression of WT Yah1^GST^ or its substitution variant was induced in the *E. coli* strain BL21(DE3) carrying the pET3aYah1-GST plasmid by addition of 1 mM IPTG at an absorbance of 0.6 at 600 nm. During overnight expression, cells were supplemented with 50 μM ferric ammonium citrate. After ∼16 h, cells were harvested and lysed in a French press in buffer I (50 mM Tris–HCl, pH 7.5, 200 mM NaCl, 1 mM PMSF, 1 mM DTT, 10% [v/v] glycerol, and 0.05% Triton X-100). After a clarifying spin, the supernatant was loaded on a 1 ml glutathione agarose column (Fluka) equilibrated with 10 volumes of buffer I. Next, the column was washed with 100 ml of buffer I and with 10 volumes of buffer I with 0.5 M NaCl and with 10 volumes of buffer I with 10 mM MgCl_2_ and 1 mM ATP. After a final wash with 10 volumes of buffer I, proteins were eluted with buffer E (50 mM Tris–HCl, pH 7.5, 200 mM NaCl, 1 mM DTT, 10% [v/v] glycerol, 0.05% Triton X-100, and 50 mM reduced glutathione). Fractions containing Yah1^GST^ were pooled, concentrated through ultrafiltration, dialyzed in order to change buffer to buffer G (50 mM Tris [pH 7.5]; 50 mM NaCl; and 10% [v/v] glycerol), and stored at −70 °C. WT or substitution variants of *S. cerevisiae* Yfh1, with a C-terminal polyhistidine tag or fusion to GST at the C terminus, Yfh1^GST^, were purified as described previously (39). Nfs1–Isd11 complex was purified from *E. coli* BL21-CodonPlus harboring plasmid pETDuet-His-NFS1–ISD11 coexpressing Nfs1 with an N-terminal polyhistidine tag and Isd11 as described previously (46). Purified Nfs1–Isd11 complex contains ACP from *E. coli* (ACP_Ec_), as revealed by mass spectrometry (MS) analysis (Fig. S17). Therefore, the Nfs1–Isd11–ACP_Ec_ complex is referred to as NIA throughout the article. Recombinant Isu1_Ct_ or Isu1_Sc_ with a polyhistidine tag at the C terminus was purified as described (41, 47). In all cases, protein concentrations, determined using the Bradford (Bio-Rad) assay with bovine serum albumin as a standard, are expressed as the concentration of monomers.

### GST pull-down assay

Pull-down experiments were performed as described (48). In short, 2.5 μM of GST-tagged bait protein (Yah1^GST^ or Yfh1^GST^ in their WT or substitution variant form; GST was used as a control for the background binding) was incubated with the indicated concentrations of NIA and/or Isu1 in buffer PD (40 mM Hepes–KOH, pH 7.5; 5% [v/v] glycerol, 100 mM KCl, 1 mM DTT, and 10 mM MgCl_2_) for 15 min at 25 °C to allow complex formation. Reduced glutathione-immobilized agarose beads were pre-equilibrated with 0.1% bovine serum albumin, 0.1% Triton X-100, and 10% (v/v) glycerol in PD buffer. About 40 μl of beads (20 μl bead volume) were added to each reaction and incubated at 4 °C for 1 h with rotation. The beads were washed three times with 500 μl of PD buffer. Proteins bound to the beads were incubated with 20 μl of twofold-concentrated Laemmli sample buffer (125 mM Tris–HCl, pH 6.8; 5% SDS, 10% 2-mercaptoethanol; 20% [v/v] glycerol) for 10 min at 100 °C, and aliquots were loaded on SDS-PAGE and visualized by InstantBlue (abcam) staining. In the case of competition experiments, the competitor (either Yah1 or Yfh1 in their WT or substitution variant version) was added to the reaction mixture at the indicated concentration.

### Densitometry analysis

Densitometry of InstantBlue stained gels was performed using the program ImageJ (49). The values obtained for each analyzed protein were normalized to the levels of bait protein (*i.e.*, Yah1^GST^, Yfh1^GST^, or GST) in each analyzed lane. After normalization, the background binding values obtained for the GST control were subtracted.

### BLI

All experiments were conducted in buffer BLI (40 mM HEPES–KOH, pH 7.5; 5% glycerol; 100 mM KCl; 1 mM DTT; 10 mM MgCl_2_; 0.05% Triton X-100) using a manual single-channel BLItz instrument (Pall ForteBio), operating at room temperature. Sensograms were recorded as a function of time. After an initial (30 s) equilibration step in BLI buffer, Yah1^GST^ or Yfh1^GST^ (at 10 μM) was immobilized on anti-GST biosensors in the presence of bovine serum albumin at 0.5 mg/ml (200 s). The sensors were then washed with BLI buffer (200 s). Following a baseline step (30 s), the sensors were immersed for 200 s into solutions containing analytes to measure association, followed by immersion for 200 s into protein-free solutions to record dissociation. To assess the background binding, sensors with immobilized GST were used. Anaerobic BLI measurements were performed in the anaerobic chamber (Coy Instruments). Prior to the anaerobic experiments, Yah1^GST^ was reduced in the presence of 5 mM sodium dithionite for 30 min. After reduction, sodium dithionite was removed from Yah1^GST^ by using a Zeba Spin Desalting Column (Thermo Fisher Scientific). To determine association rate constant (*k*_*a*_) and *k*_*d*_ rate constant as well as *K*_*D*_, data were fitted with binding models available in the Octet Data Analysis HT 12.0 software. In all cases, the best fit was obtained for the 2:1 heterogenous ligand model.

### CD measurements

Measurements of CD spectra in the UV range (196–260 nm) were performed on a Jasco J-1500 CD Spectrometer with a 1 nm step size at 20 °C. The protein concentration was 15 μM for WT or substitution variants of Yah1. Measurements were performed in buffer CD (20 mM potassium phosphate, pH 8.0, and 80 mM KCl) in a quartz cuvette with a path length of 1 mm. Spectra were measured in millidegrees (mdeg), corrected for buffer effects, and converted to mean residue ellipticity (Θ). CD measurements in the visible range (350–650 nm) were performed in buffer FS (25 mM Tris–HCl, pH 7.5; 50 mM NaCl; 10% glycerol) at 20 °C in a quartz cuvette with a path length of 10 mm. The protein concentration was 30 μM for WT or substitution variants of Yah1. Yah1 was chemically reduced using 5 mM sodium dithionite. Spectra were measured in mdeg and corrected for buffer effects. *T*_M_ of proteins was measured as follows. A wavelength corresponding to the maximum at 432 nm for Yah1 was used to monitor the thermal melting, expressed as a change in mdeg as the temperature increased from 20 to 95 °C at a rate of 0.5 °C/min. Data were analyzed using Spectra Manager, version 2, JASCO software in order to calculate the *T*_*M*_ values for each protein.

### MS

Because ACP_Ec_, which has a molecular weight of 8.6 kD is not easily visible upon staining after gel electrophoresis, we carried out MS analysis to confirm its presence in our preparation of the NIA complex. We also used MS to identify degradation products contaminating our preparation of Yah1^GST^ fusion protein. To this end, we used previously characterized (39) Yfh1^GST^–NIA–Isu1 complex separated on 4 to 16% gradient SDS-PAGE. Gel pieces containing ACP_Ec_ were washed with 25 mM NH_4_HCO_3_ and with 50% acetonitrile in 25 mM NH_4_HCO_3_ to remove Coomassie Brilliant Blue G-250 staining. Next, the gels were shrunk with acetonitrile and subjected to reduction with 10 mM DTT in 100 mM NH_4_HCO_3_ for 30 min at 57 °C. Free cysteines were alkylated with 0.5 M iodoacetamide in 100 mM NH_4_HCO_3_ (45 min in a dark room at room temperature), and proteins were digested in-gel overnight with 10 ng/μl trypsin (catalog no.: V5280; Promega) in 25 mM NH_4_HCO_3_. After digestion, the peptides were extracted from the gel by sequential washing with a solution of 25 mM NH_4_HCO_3_ (one time) and 10% formic acid in water (two times). The gels were finally shrunk with 100% acetonitrile, and the resulting solutions were combined, evaporated to dryness on a SpeedVac, then dissolved in 1% acetic acid with water, and desalted according to the StageTips C18 procedure (50).

LC/MS–MS analysis was performed in the Mass Spectrometry Laboratory (Intercollegiate Faculty of Biotechnology University of Gdansk and Medical University of Gdansk), using a TripleTOF 5600+ hybrid mass spectrometer with a DuoSpray Ion Source (AB SCIEX) connected with an Ekspert MicroLC 200 Plus System (Eksigent). Peptides were separated by a 28 min gradient of 10 to 90% solvent B in solvent A, with flow rate of 10 μl/min, followed by 2 min regeneration and re-equilibration. Solvents A and B consisted of 0.1% (v/v) formic acid in water and acetonitrile, respectively. Column parameters: Exigent microLC column ChromXP C18CL (3 μm, 120 Å, 150 × 0.3 mm) and an injection volume of 5 μl. The microLC–MS/MS system was controlled by the AB SCIEX Analyst TF 1.6 software. The data-dependent acquisition experiments were conducted for all the investigated samples. The TOF MS survey scan was performed in the *m/z* range of 100 to 2000 for 50 ms.

Data were searched with PEAKS Studio 10.6 software (51) using PEAKS standard protocol (*de novo* + PEAKS DB + PEAKS PTM + SPIDER) based on UniProtKB SwissProt database (*E. coli* and *S. cerevisiae*, retrieval dates: October 12, 2020 and October 26, 2020; number of entries searched: 1,759,377 and 84,397, respectively), with the following parameters: enzyme: trypsin with [D|P], digest mode: specific, parent ions mass error tolerance: 0.1 Da, fragment ion mass error tolerance: 0.2 Da, max missed cleavages: 3, fixed modifications: Carbamidomethyl (C), variable modifications: Acetylation (N-term), Oxidation (M). Protein identification was validated using a target-decoy search strategy with 1% false discovery rate threshold.

### Ferredoxin phylogeny

Ferredoxin sequences orthologous to bacterial Fdx, yeast Yah1, mammalian FDX1 and FDX2 were retrieved from OMA (*o*rthologous *ma*trix) orthology database (respective OMA groups: 0679456, 0310453, 0459198, and 0508748) (52). Because the FDX1 and FDX2 orthology groups contained few proteins from species outside chordates, the analysis was extended to other animal groups using a probabilistic profile search. To this end, an initial set of FDX1 and FDX2 orthologs from OMA was aligned using Clustal Omega, version 1.2.4 (53) with default parameters and converted into hidden Markov model (HMM) profiles using hmmbuild program from HMMER package (54). To identify FDX1 and FDX2 homologs, hmmsearch was performed with an e-value cutoff >10^−3^ using full protein sets retrieved from UniProt database (55). Best hits identified as FDX1 or FDX2 orthologs were added to the sequence dataset (Supporting information; sequences). Sequences were realigned and trimmed manually; nonorthologous sequences were removed. About 1000 maximum likelihood searches were performed using IQ-TREE (56) with 1000 ultrafast bootstrap replicates, using the LG model of amino acid substitution, gamma model of rate heterogeneity with four discrete rate categories, and the estimate of proportion of invariable sites (LG + I + G4). Best-fit model was determined by ModelFinder in IQ-TREE. Alignment logo was generated using the WebLogo server (57).

### Computational model of NIA–Isu1–Yah1 complex

To obtain the computational model of NIA and NIA–Isu1 complexes, we used homology models of yeast Nfs1 and Isd11 from SwissModel repository (P25374 and Q6Q560, respectively), ACP_Ec_ structure (PDB: 6NZU), and Isu1 model obtained using I-TASSER (58), based on the X-ray structure of the IscS–IscU complex (PDB: 3LVL). The NIA and NIA–Isu1 complexes were modeled by superimposition of proteins listed previously on the cryo-EM structure of human frataxin-bound NIA complex (PDB: 6NZU) using visual molecular dynamics (59). Next, the crystal structure of Yah1 (PDB: 2MJD) was docked to these complexes using ClusPro (60), without restraints on protein–protein interactions. Side chains were then relaxed, and minor steric clashes were resolved during energy minimization in GROMACS 5.1.7 (61), according to the minimization protocol described (62).

## Data availability

The MS proteomics data have been deposited to the ProteomeXchange Consortium *via* the PRIDE partner repository with the dataset identifier PXD029519 and 10.6019/PXD029519. Additional data available upon request, please e-mail Dr Rafal Dutkiewicz (rafal.dutkiewicz@ug.edu.pl).

## Supporting information

This article contains supporting information.

## Conflict of interest

The authors declare that they have no conflicts of interests with the content of this article.
