# Supplementary material for: During FeS cluster biogenesis, ferredoxin and frataxin use overlapping binding sites on yeast cysteine desulfurase Nfs1
Source: J Biol Chem. 2022 Jan 11;298(2):101570. doi: 10.1016/j.jbc.2022.101570 (PMC8888459; doi:10.1016/j.jbc.2022.101570)
Supplement: Supplemental Figures S1–S17 [file mmc1.pdf]

During FeS-cluster biogenesis ferredoxin and frataxin use overlapping binding sites on yeast cysteine desulfurase Nfs1

Marta A. Uzarska, Igor Grochowina, Joanna Soldek, Marcin Jelen, Brenda Schilke, Jaroslaw Marszalek, Elizabeth A. Craig, Rafal Dutkiewicz

Supporting information

Page

Figure S1. Circular dichroism (CD) spectra of purified Yah1 and Yah1^{GST} S-1

Figure S2. *In vivo* tests of Yah1^{GST} fusion and variants. S-2

Figure S3. Mass-spectrometry (MS) identification of degradation products present in the Yah1^{GST} protein preparation S-3

Figure S4. SDS-PAGE gels for Fig. 1E. S-4

Figure S5. Kinetics analysis for the bio-layer interferometry (BLI) results from Fig. 1F. S-5

Figure S6. Bio-layer interferometry (BLI) analysis of interaction between un-tagged Yah1 and immobilized NIA complex. S-6

Figure S7. SDS-PAGE gels for Fig. 2D, F. S-7

Figure S8. Kinetics analysis for the bio-layer interferometry (BLI) results from Fig. 2E. S-8

Figure S9. Kinetics analysis for the bio-layer interferometry (BLI) results from Fig. 2G. S-9

Figure S10. Kinetics analysis for the bio-layer interferometry (BLI) results from Fig. 3C. S-10

Figure S11. Maximum-likelihood phylogeny of ferredoxin orthologs. S-11

Figure S12. CD spectra of purified Yah1 WT and Yah1(D128K,D131K). S-12

Figure S13. Kinetics analysis for the bio-layer interferometry (BLI) results from Fig. 5C. S-13

Figure S14. Kinetics analysis for the bio-layer interferometry (BLI) results from Fig. 5F. S-14

Figure S15. Kinetics analysis for the bio-layer interferometry (BLI) results from Fig. 6A. S-15

Figure S16. Kinetics analysis for the bio-layer interferometry (BLI) results from Fig. 6C. S-16

Figure S17. Mass-spectrometry (MS) identification of ACP_{Ec} in the preparation of NIA complex. S-117

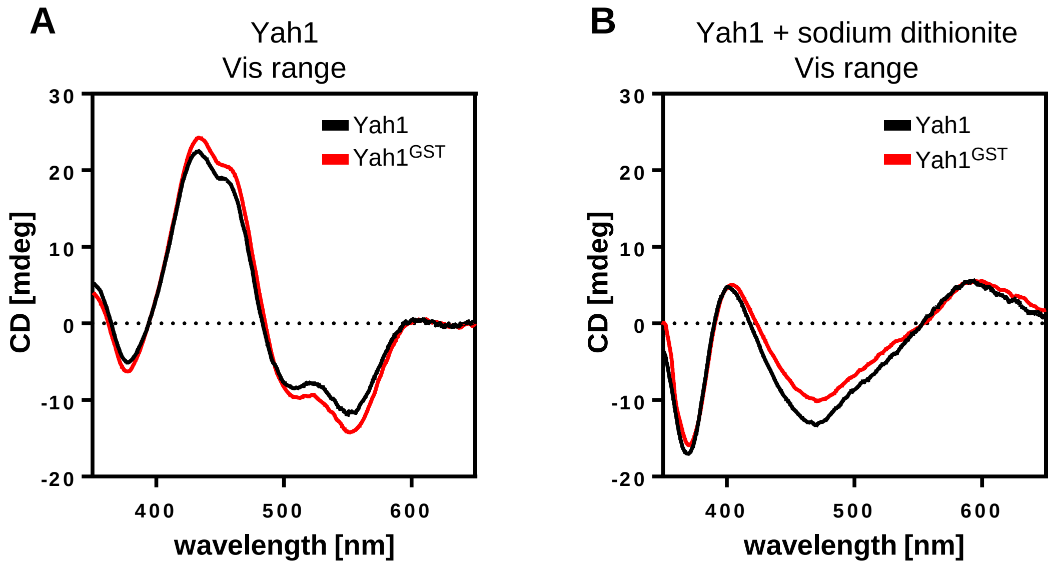

Fig. S1 Circular dichroism (CD) spectra of purified Yah1 and Yah1^{GST}. Visible range CD spectra of 30 μM Yah1 and Yah1^{GST} were measured before (**A**) and after chemical reduction with 5 mM sodium dithionite (**B**), as described in the Experimental procedures.

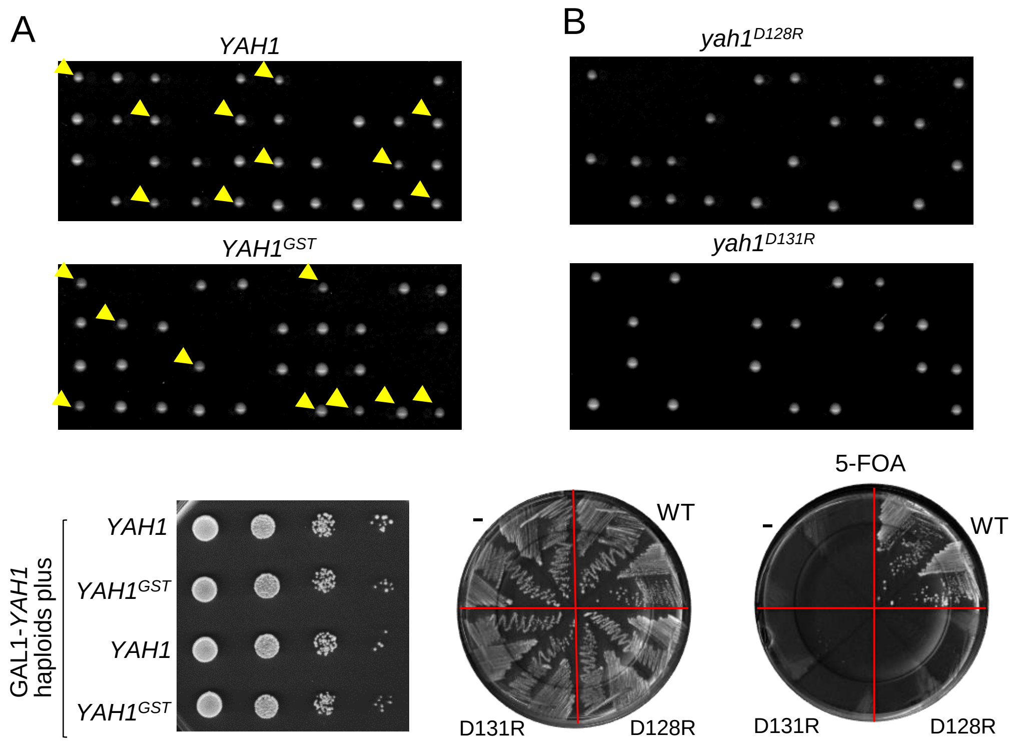

Fig. S2 ***In vivo*** **tests of Yah1^{GST} fusion and variants.** (**A,B**) (**Top**) A heterozygous diploid *S. cerevisiae* strain (*YAH1*/GAL1-*YAH1* (*HIS3*)) carrying WT *YAH1* driven by the native promoter on one chromosome and driven by the *GAL1* promoter on the other chromosome, that is tagged with the *HIS3* auxotrophic marker, was transformed with plasmids containing: *YAH1*, *YAH1*^{GST} fusion, *yah1*^{D128R} or *yah1*^{D131R}. Resulting transformants were sporulated and the resulting asci dissected onto rich glucose media which turns off expression of GAL1 driven *YAH1*. (**A**) Dissection plates were replica plated onto His omission media to test for the presence of the GAL1-driven *YAH1*, with growth indicated by yellow arrowhead. (**B**) Growth of only 2 spores in each tetrad (none were His+, thus none contained GAL1-*YAH1*), which indicates the inability of Yah1^{D128R} and Yah1^{D131R} to support growth. (**Bottom**) (**A**) 10-fold serial dilutions of GAL1-driven *YAH1* haploids containing plasmids expressing either *YAH1* or *YAH1*^{GST} (representative colonies marked by yellow arrowheads on dissection plates at top) were plated on His omission glucose-based media and incubated at 30°C for 2 days. (**B**) Since no growth was observed on the dissection plates of GAL1-driven *YAH1* haploids with the two yah1 mutants, a separate experiment was carried out. GAL1-*YAH1* haploid strains carrying two plasmids, one expressing *YAH1*^{GST} and the *URA3* gene and the other expressing either *YAH1* (WT), *yah1*^{D128R} (D128R), *yah1*^{D131R} (D131R) or no Yah1 gene (-) were streaked onto glucose media either lacking (left) or containing 5-fluororotic acid (5-FOA) (right). 5-FOA is toxic to cells expressing Ura3 – thus only those transformants expressing functional Yah1 from the non *URA3*-based plasmid can grow. Plates were incubated for 2 days at 30°C.

A

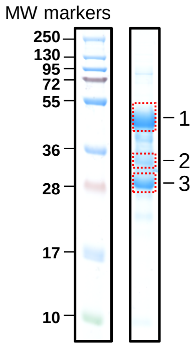

B

1	-10lgP	Coverage (%)	Peptides	Unique peptides
GST	375.91	91	39	39
Yah1	153.38	30	5	5

2	-10lgP	Coverage (%)	Peptides	Unique peptides
GST	287.88	80	37	37
Yah1	76.54	21	3	3

3	-10lgP	Coverage (%)	Peptides	Unique peptides
GST	470.83	91	41	41

Fig. S3 Mass-spectrometry identification of degradation products present in the Yah1^{GST} protein preparation. (**A**) SDS-PAGE gel of Yah1^{GST} protein preparation (4 μg) used in the pull-down and BLI experiments; positions of bands analyzed by mass spectrometry (MS) are indicated. (**B**) Identification of peptides detected in LC-MS/MS analysis of the excised bands was performed in PEAKS Studio 10.6 software, using PEAKS standard protocol, based on *E. coli* protein sequences from UniProtKB database (retrieval date: 12.10.2020; 1759377 entries searched) and Yah1^{GST} fusion protein sequence, with parameters as described in the Experimental procedures.
PEAKS significance score (-10lgP), coverage of protein sequences with peptides, and number of peptides detected are indicated.

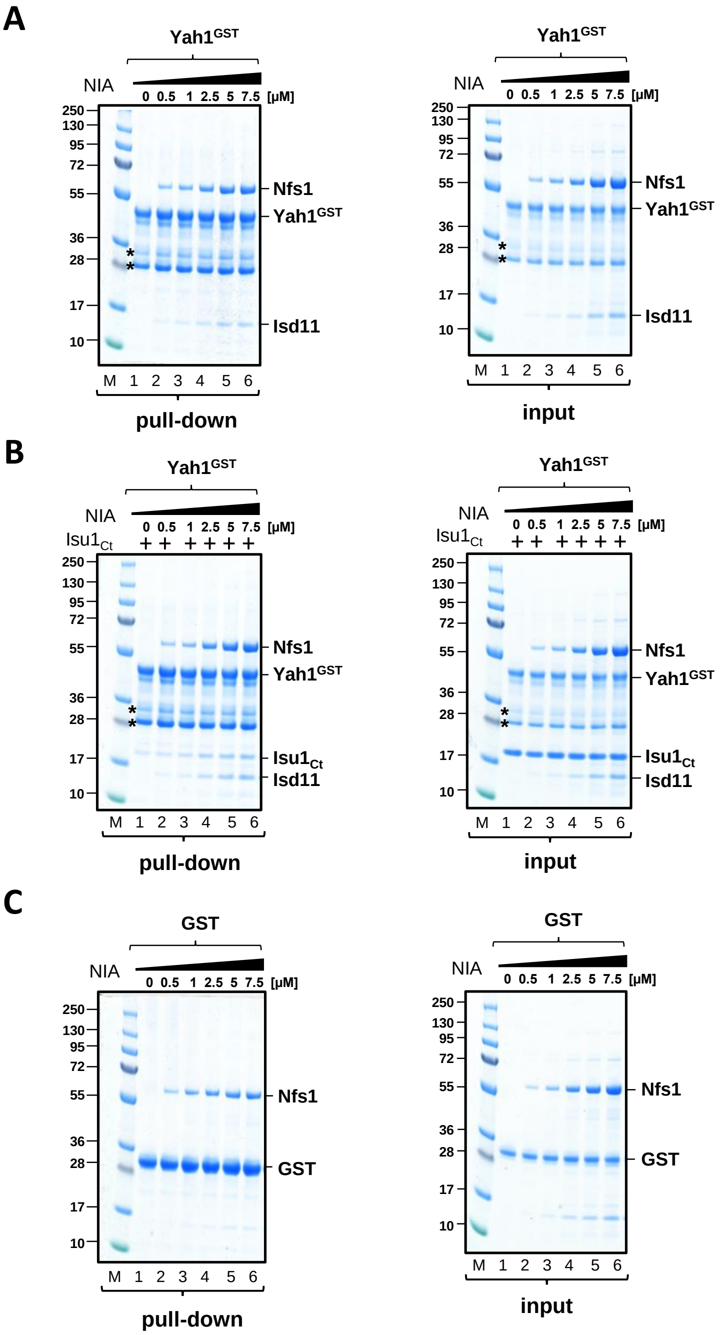

Fig. S4 SDS-PAGE gels for Fig. 1E. Yah1^{GST} (**A,B**) or GST (**C**) (at 2.5 μM) (background control) were incubated with increasing concentrations of NIA in the absence (**A**) or presence (**B**) of Isu1$_{Ct}$ (7.5 μM). Glutathione resin was added to pull-down GST and associated proteins, which were then separated by SDS-PAGE and stained with 'Instant Blue'. 'M' lanes having molecular weight markers. Input- loading controls- 5% of the reaction volume. Asterisks mark the Yah1^{GST} degradation products (see Fig. S3).

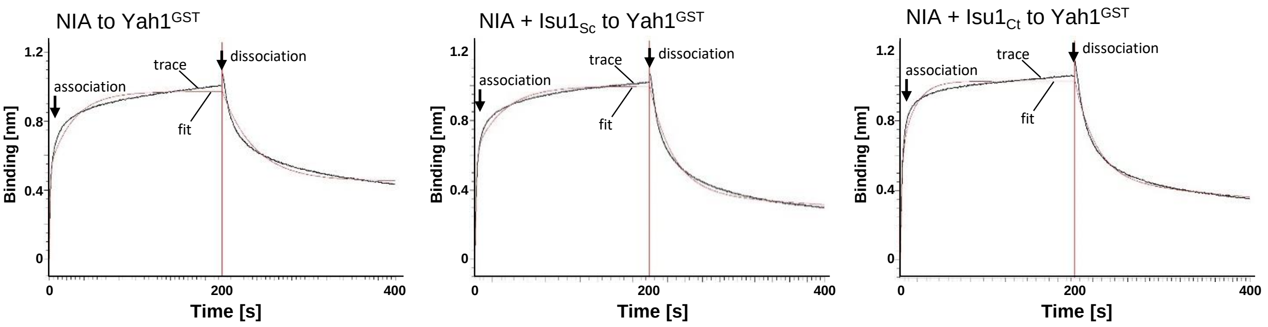

	K_{D1} [M]	K_{D2} [M]	k_{a1} [$M^{-1}s^{-1}$]	k_{a2} [$M^{-1}s^{-1}$]	k_{d1} [s^{-1}]	k_{d2} [s^{-1}]	% K_{D1}	% K_{D2}	R^2
**NIA to Yah1^{GST}**	9.66e-11	2.8e-07	3.66e+03	9.55e+04	3.54e-07	2.67e-02	46	54	0.988
**NIA + Isu1$_{Sc}$ to Yah1^{GST}**	3.25e-07	6.6e-07	3.00e+03	5.7e+04	9.77e-04	3.75e-02	38	62	0.996
**NIA + Isu1$_{Ct}$ to Yah1^{GST}**	1.84e-07	8.0e-07	6.39e+03	5.0e+04	1.18e-03	4.03e-02	43	57	0.995

Fig. S5 Kinetics analysis for the bio-layer interferometry (BLI) results from Fig. 1F. A 2:1 heterogenous ligand interaction model was fit to the BLI traces from Fig. 1F (each trace line (black) is on a separate plot for clarity). Two sets of association and dissociation rate constants (k_{a1}, k_{d1} and k_{a2}, k_{d2}) were derived from each fit. Based on their values two equilibrium binding constants ($K_{D1,}$ K_{D2}) and their contributions to the interaction (%$K_{D1,}$ %K_{D2}) were calculated. The R^2 value indicates how well the fit and the experimental data correlate.

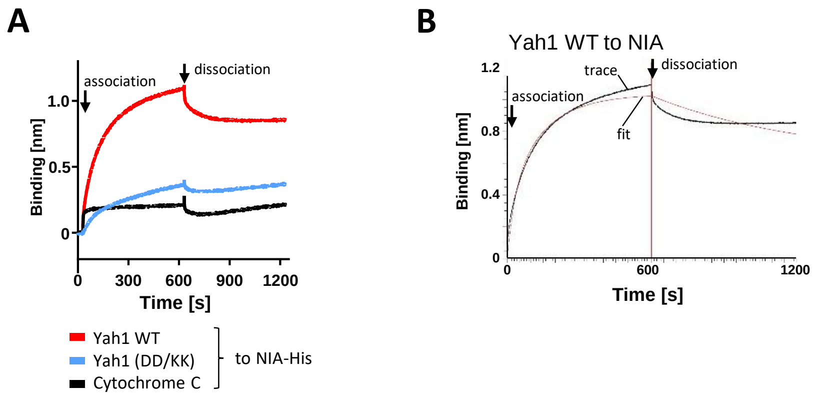

	K_{D1} [M]	K_{D2} [M]	k_{a1} [$M^{-1}s^{-1}$]	k_{a2} [$M^{-1}s^{-1}$]	k_{d1} [s^{-1}]	k_{d2} [s^{-1}]	% K_{D1}	% K_{D2}	R^2
**Yah1 WT**	3.07e-10	5.44e-07	6.75e+02	2.87e+03	2.08e-07	1.56e-03	61	39	0.931

Fig. S6 Bio-layer interferometry (BLI) analysis of interaction between un-tagged Yah1 and immobilized NIA complex. (**A**) Association phase - Ni-NTA sensors loaded with Nfs1-His in NIA complex (1 μM) were immersed at 30 s time point into solution containing untagged Yah1 WT, Yah1(D128K,D131K) or Cytochrome C (background control) at (10 μM). Dissociation phase - at 600 s the sensors were placed in solution without proteins. (**B**) A 2:1 heterogenous ligand interaction model was fit to the BLI trace (black line) representing Yah1 WT interaction with the immobilized NIA complex. Two sets of association and dissociation rate constants (k_{a1}, k_{d1} and k_{a2}, k_{d2}) were derived from this fit. Based on their values two equilibrium binding constants ($K_{D1,}$ K_{D2}) and their contributions to the interaction (%$K_{D1,}$ %K_{D2}) were calculated. The R^2 value indicates how well the fit and the experimental data correlate.

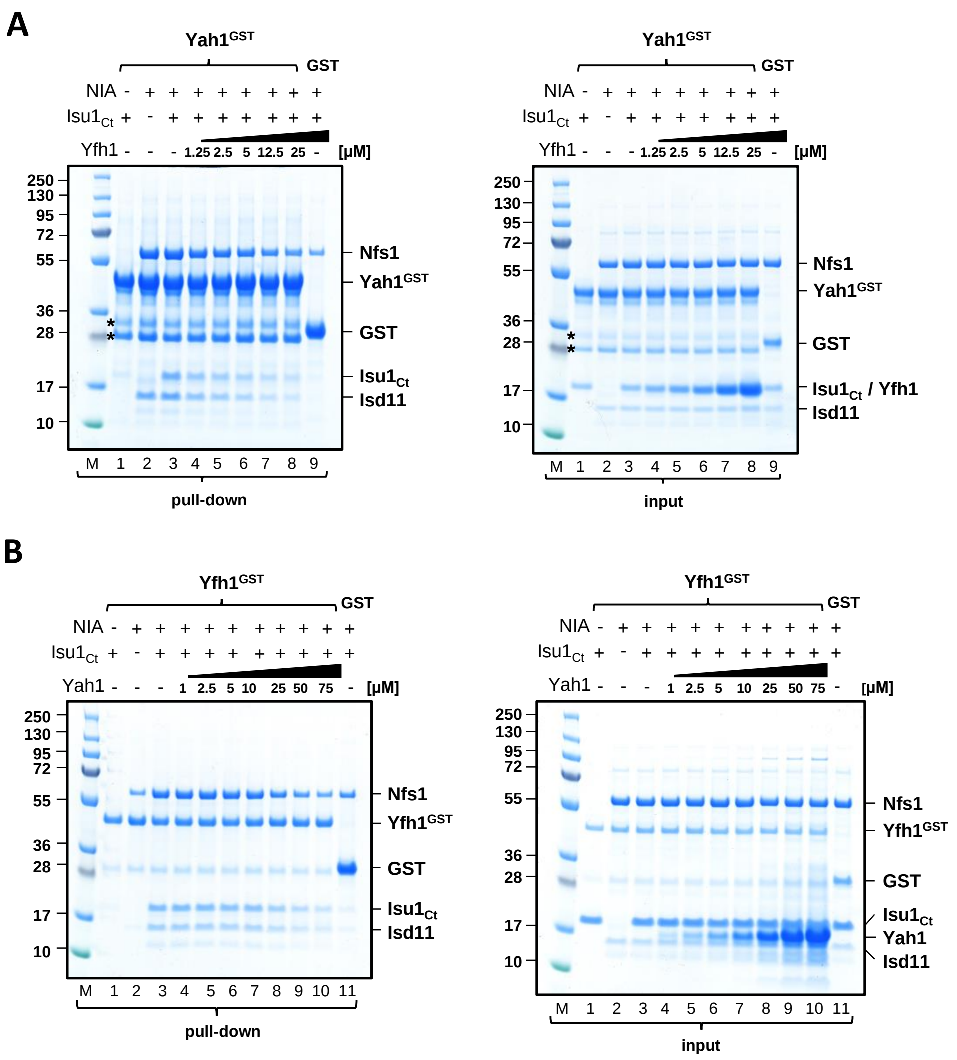

Fig. S7 SDS-PAGE gels for Fig. 2D, F. (**A**) $Yah1^{GST}$ (2.5 μM) or GST (2.5 μM) (background control) were incubated with NIA (5 μM) and $Isu1_{Ct}$ (7.5 μM) to allow complex formation. Then increasing concentrations of Yfh1 were added to the reaction mixtures. Glutathione resin was added to pull-down GST and associated proteins, which were then separated by SDS-PAGE and stained with 'Instant Blue'. 'M' lanes having molecular weight markers. Input- loading controls- 5% of the reaction volume. Asterisks mark the $Yah1^{GST}$ degradation products (Fig. S3). (**B**) $Yfh1^{GST}$ (2.5 μM) or GST (2.5 μM) (background control) were incubated with NIA (5 μM) and $Isu1_{Ct}$ (7.5 μM) to allow complex formation. Then increasing concentrations of Yah1 were added to the reaction mixtures. Reactions were treated as described in A.

A

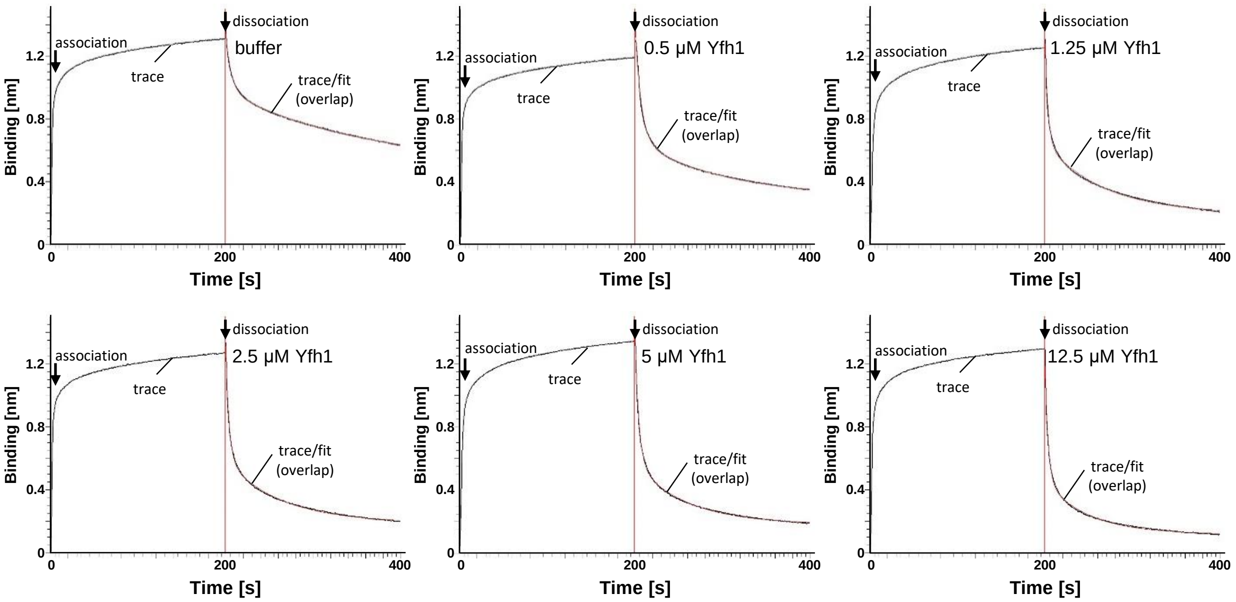

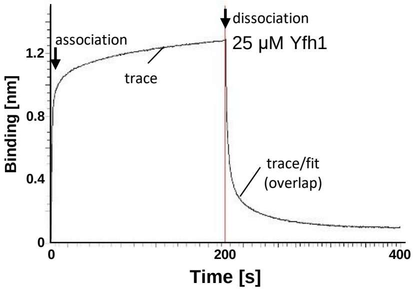

Dissociation:	k_{d1} [s^{-1}]	k_{d2} [s^{-1}]	R^2
**buffer**	6.53e-03	1.57e-01	0.999
**0.5 μM Yfh1**	9.97e-03	1.46e-01	0.998
**1.25 μM Yfh1**	1.48e-02	2.93e-01	0.997
**2.5 μM Yfh1**	1.60e-02	2.59e-01	0.998
**5 μM Yfh1**	1.73e-02	2.25e-01	0.998
**12.5 μM Yfh1**	2.04e-02	2.80e-01	0.997
**25 μM Yfh1**	2.96e-02	3.79e-01	0.997

B

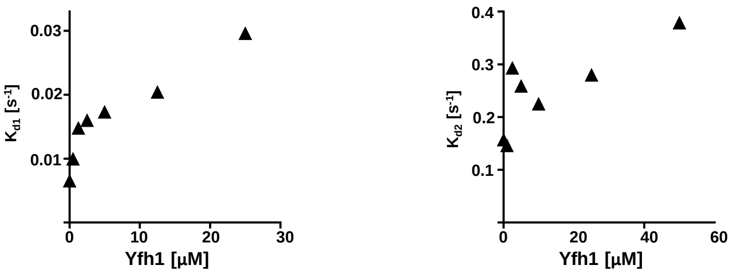

Fig. S8 Kinetics analysis for the bio-layer interferometry (BLI) results from Fig. 2E. (**A**) A 2:1 heterogenous ligand interaction model was fit to the dissociation phase of the BLI traces from Fig. 2E (each trace is on a separate plot for clarity; note that the trace line and the fit line overlap). From each fit two dissociation rate constants (k_{d1} and k_{d2}) were derived. The R^2 value indicates how well the fit and the experimental data correlate. (**B**) The values of k_{d1} and k_{d2} were plotted against the competitor (Yfh1) concentration.

A

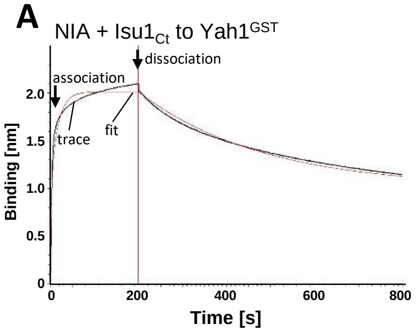

K_{D1} [M]	K_{D2} [M]	k_{a1} [$M^{-1}s^{-1}$]	k_{a2} [$M^{-1}s^{-1}$]	k_{d1} [s^{-1}]	k_{d2} [s^{-1}]	% K_{D1}	% K_{D2}	R^2
5.34e-11	6.39e-08	6.17e+03	5.54e+04	3.29e-07	3.54e-03	50	50	0.987

B

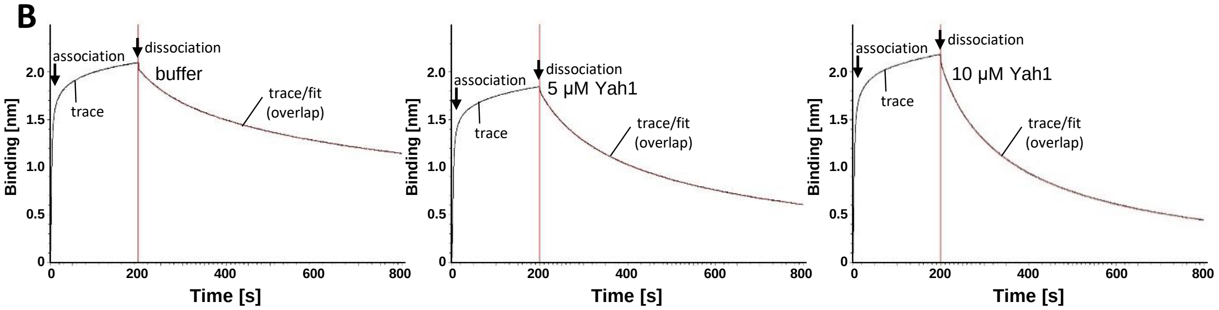

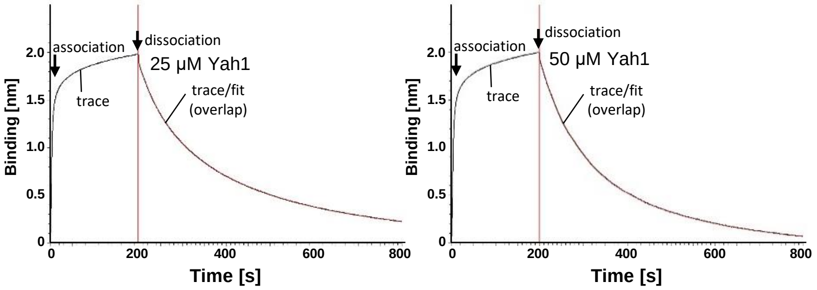

Dissociation:	k_{d1} [s^{-1}]	k_{d2} [s^{-1}]	R^2
**buffer**	2.20e-03	1.59e-02	0.999
**5 μM Yah1**	2.77e-03	1.61e-02	0.999
**10 μM Yah1**	3.51e-03	1.90e-02	0.999
**25 μM Yah1**	3.72e-03	1.86e-02	0.999
**50 μM Yah1**	3.59e-03	1.38e-02	0.999

C

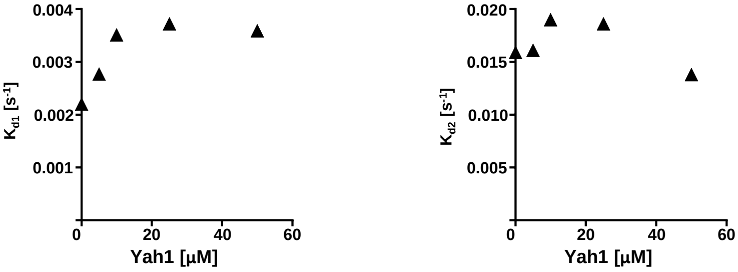

Fig. S9 Kinetics analysis for the bio-layer interferometry (BLI) results from Fig. 2G. (**A**) A 2:1 heterogenous ligand interaction model was fit to the BLI trace (black line) of NIA + $Isu1_{Ct}$ binding to immobilized $Yfh1^{GST}$ from Fig. 2G. Two sets of association and dissociation rate constants (k_{a1}, k_{d1} and k_{a2}, k_{d2}) were derived from this fit. Based on their values two equilibrium binding constants ($K_{D1,}$ K_{D2}) and their contributions to the interaction (%$K_{D1,}$ %K_{D2}) were calculated. The R^2 value indicates how well the fit and the experimental data correlate. (**B**) A 2:1 heterogenous ligand interaction model was fit to the dissociation phase of the BLI traces (black lines) from Fig. 2G (each trace is on a separate plot for clarity; note that the fit line and the trace line overlap). Two dissociation rate constants (k_{d1} and k_{d2}) were derived from each fit. The R^2 value indicates how well the fit and the experimental data correlate. (**C**) The values of k_{d1} and k_{d2} were plotted against the competitor (Yah1) concentration.

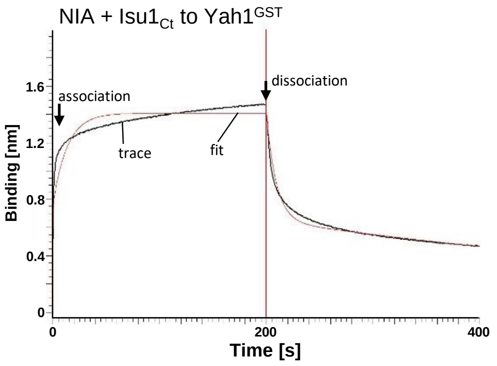

	K_{D1} [M]	K_{D2} [M]	k_{a1} [$M^{-1}s^{-1}$]	k_{a2} [$M^{-1}s^{-1}$]	k_{d1} [s^{-1}]	k_{d2} [s^{-1}]	% K_{D1}	% K_{D2}	R^2
**NIA + Isu1$_{Ct}$ to Yah1^{GST}**	2.28e-07	2.12e-07	7.33e+03	5.12e+05	1.67e-03	1.09e-01	46	54	0.989

Fig. S10 Kinetics analysis for the bio-layer interferometry (BLI) results from Fig. 3C. A 2:1 heterogenous ligand interaction model was fit to the BLI trace (black line) representing NIA + Isu1$_{Ct}$ binding to immobilized Yah1^{GST} from Fig. 3C. Two sets of association and dissociation rate constants (k_{a1}, k_{d1} and k_{a2}, k_{d2}) were derived from this fit. Based on their values two equilibrium binding constants ($K_{D1,}$ K_{D2}) and their contribution to the interaction (%$K_{D1,}$ %K_{D2}) were calculated. The R^2 value indicates how well the fit and the experimental data correlate.

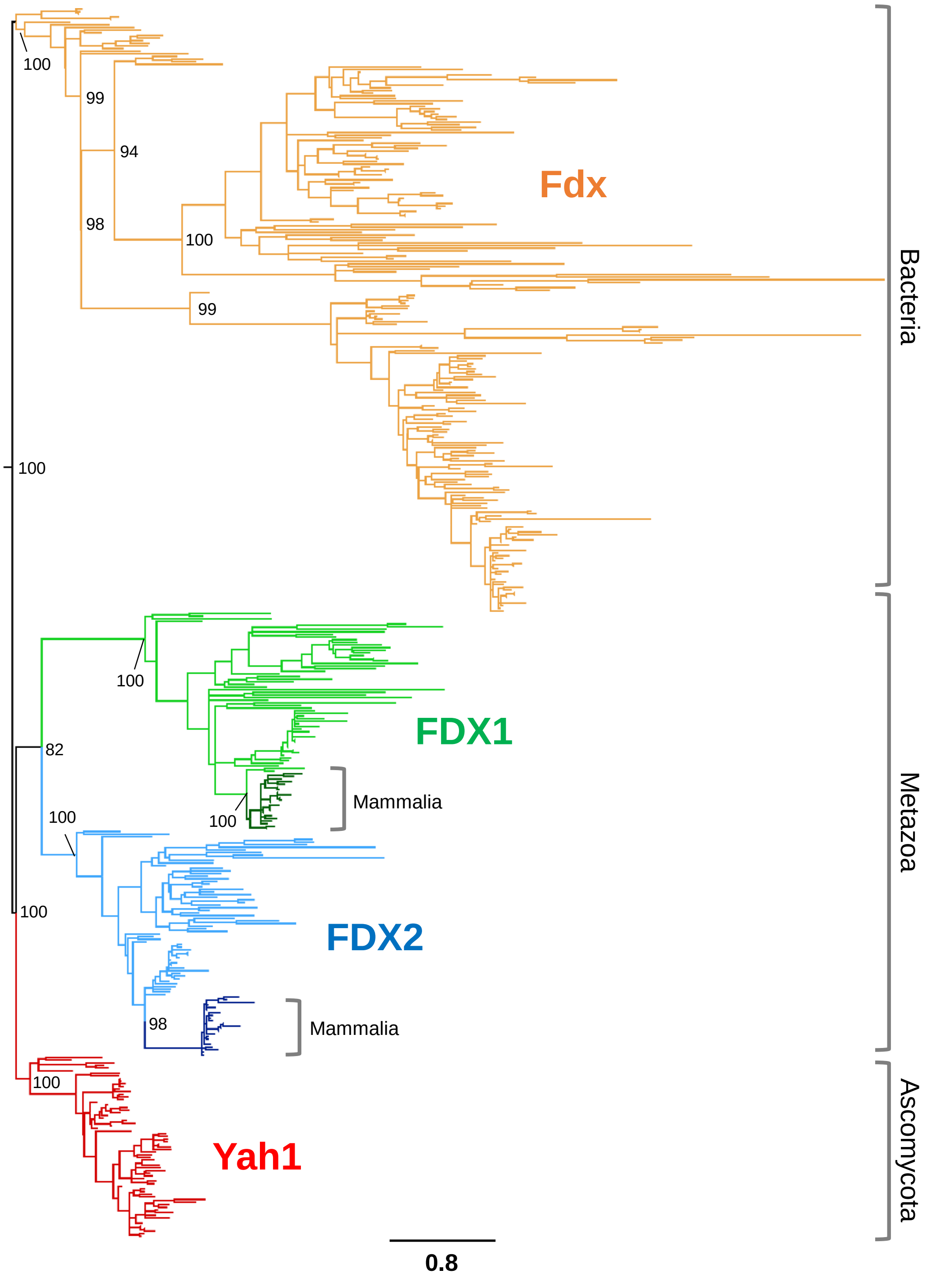

Fig. S11 Maximum-likelihood phylogeny of ferredoxin orthologs. Ferredoxin orthologs from Bacteria (Fdx- orange), Metazoa (FDX1 and FDX2- green and blue, respectively) and Ascomycota (Yah1- red) are marked on the tree; Mammalian FDX1 and FDX2 are depicted by darker green and blue colors. Bootstrap support for major clades is indicated. Scale is in amino acid substitutions per site. The tree was rooted between bacterial and eukaryotic sequences.

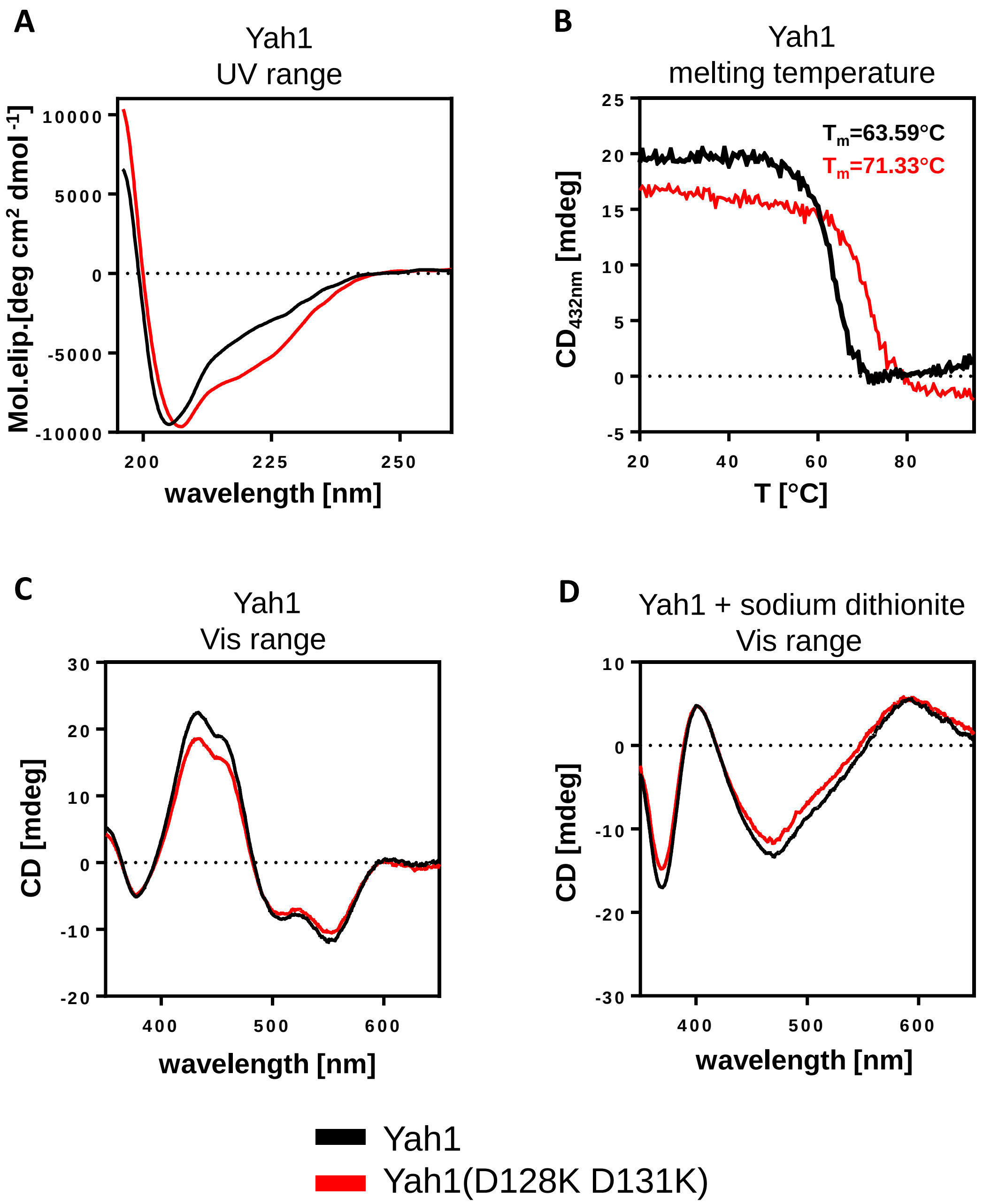

Fig. S12 CD spectra of purified Yah1 WT and Yah1(D128K,D131K). (**A**) CD spectra of 30 μM Yah1 WT and Yah1(D128K,D131K) were measured in the UV range. (**B**) Melting temperatures of Yah1 WT and Yah1(D128K,D131K) were determined as described in the Experimental procedures. (**C,D**) CD spectra of 30 μM Yah1 WT and Yah1(D128K, D131K) were measured in the visible range before (**C**) and after chemical reduction with 5 mM sodium dithionite (**D**), as described in the Experimental procedures.

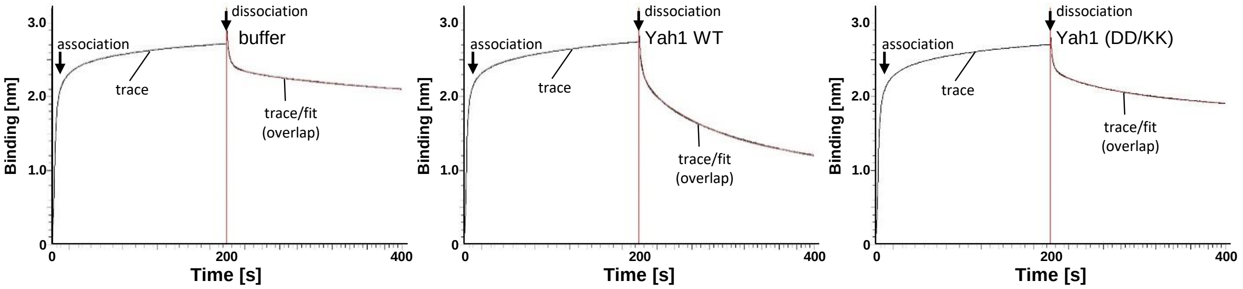

Dissociation:	k_{d1} [s^{-1}]	k_{d2} [s^{-1}]	R^2
**buffer**	7.36e-03	3.28e-01	0.998
**Yah1 WT**	1.01e-02	2.28e-01	0.999
**Yah1 (DD/KK)**	1.01e-02	3.56e-01	0.999

Fig. S13 Kinetics analysis for the bio-layer interferometry (BLI) results from Fig. 5C. A 2:1 heterogenous ligand interaction model was fit to the dissociation phase of the BLI traces (black lines) from Fig. 5C (each trace is on a separate plot for clarity; note that the trace line and the fit line overlap). From each fit two dissociation rate constants (k_{d1} and k_{d2}) were derived. The R^2 value indicates how well the fit and the experimental data correlate.

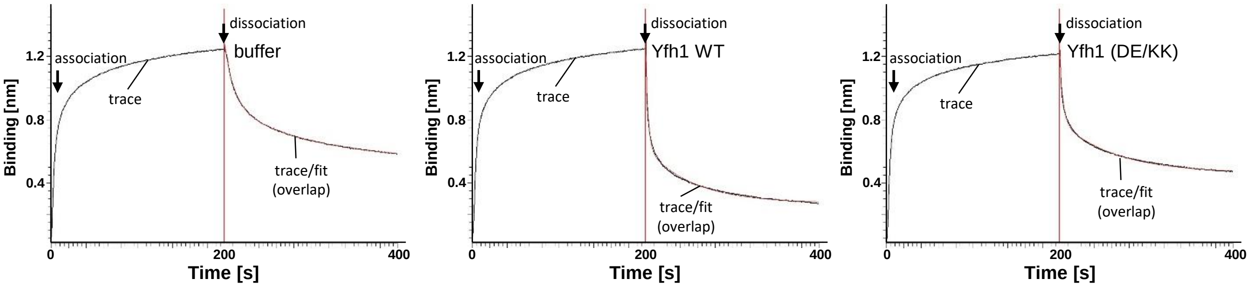

Dissociation:	k_{d1} [s^{-1}]	k_{d2} [s^{-1}]	R^2
**buffer**	1.05e-02	9.00e-02	0.999
**Yfh1 WT**	1.87e-02	3.56e-01	0.996
**Yfh1 (DE/KK)**	1.60e-02	2.69e-01	0.998

Fig. S14 Kinetics analysis for the bio-layer interferometry (BLI) results from Fig. 5F. A 2:1 heterogenous ligand interaction model was fit to the dissociation phase of the BLI traces (black lines) from Fig. 5F (each trace is on a separate plot for clarity; note that the trace line and the fit line overlap). From each fit two dissociation rate constants (k_{d1} and k_{d2}) were derived. The R^2 value indicates how well the fit and the experimental data correlate.

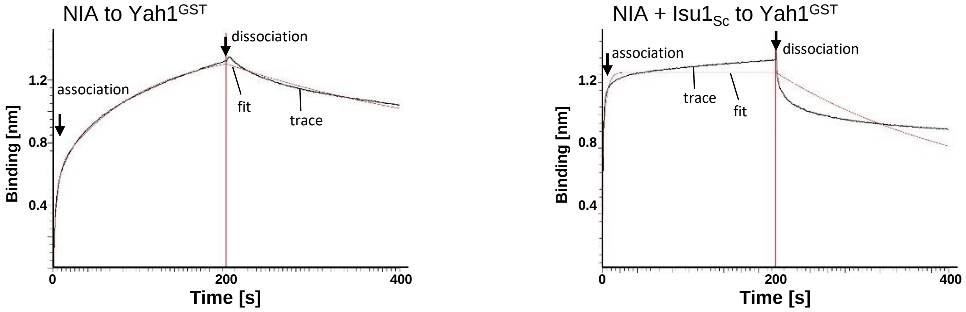

	K_{D1} [M]	K_{D2} [M]	k_{a1} [$M^{-1}s^{-1}$]	k_{a2} [$M^{-1}s^{-1}$]	k_{d1} [s^{-1}]	k_{d2} [s^{-1}]	% K_{D1}	% K_{D2}	R^2
**NIA to Yah1^{GST}**	8.20e-10	1.14e-07	4.72e+02	1.80e+04	3.88e-07	2.04e-03	61	39	0.988
**NIA + Isu1$_{Sc}$ to Yah1^{GST}**	1.65e-11	1.53e-08	2.46e+04	2.75e+05	4.05e-07	4.21e-03	37	63	0.853

Fig. S15 Kinetics analysis for the bio-layer interferometry (BLI) results from Fig. 6A. A 2:1 heterogenous ligand interaction model was fit to the BLI traces (black lines) from Fig. 6A (each trace is on a separate plot for clarity). Two sets of association and dissociation rate constants (k_{a1}, k_{d1} and k_{a2}, k_{d2}) were derived from each fit. Based on their values two equilibrium binding constants ($K_{D1,}$ K_{D2}) and their contributions to the interaction (%$K_{D1,}$ %K_{D2}) were calculated. The R^2 value indicates how well the fit and the experimental data correlate.

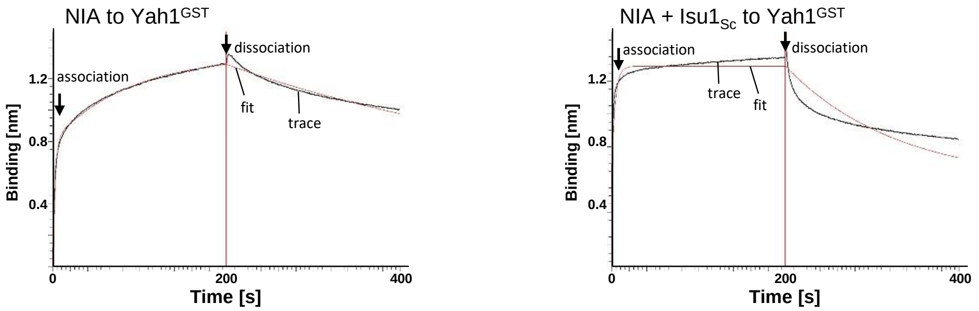

	K_{D1} [M]	K_{D2} [M]	k_{a1} [$M^{-1}s^{-1}$]	k_{a2} [$M^{-1}s^{-1}$]	k_{d1} [s^{-1}]	k_{d2} [s^{-1}]	% K_{D1}	% K_{D2}	R^2
**NIA to Yah^{GST}**	4.46e-10	6.58e-08	1.10e+03	3.76e+04	4.91e-07	2.48e-03	40	60	0.988
**NIA + Isu1$_{Sc}$ to Yah1^{GST}**	2.00e-11	1.92e-08	2.49e+04	4.04e+05	4.99e-07	7.77e-03	45	55	0.906

Fig. S16 Kinetics analysis for the bio-layer interferometry (BLI) results from Fig. 6C. A 2:1 heterogenous ligand interaction model was fit to the BLI traces (black lines) from Fig. 6C (each trace is on a separate plot for clarity). Two sets of association and dissociation rate constants (k_{a1}, k_{d1} and k_{a2}, k_{d2}) were derived from each fit. Based on their values two equilibrium binding constants ($K_{D1,}$ K_{D2}) and their contributions to the interaction (%$K_{D1,}$ %K_{D2}) were calculated. The R^2 value indicates how well the fit and the experimental data correlate.

A

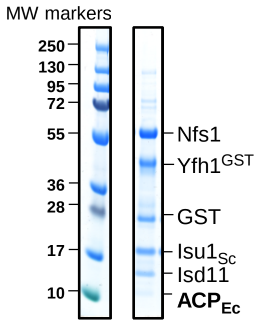

B

Protein name	Organism	Uniprot ID	-10lgP	Coverage (%)	Peptides	Unique peptides	Mass [kDa]
**ACP**	*E.coli*	P0A6A8	106.82	33	3	1	8.6

Fig. S17 Mass-spectrometry (MS) identification of ACP_{Ec} in the preparation of NIA complex. (**A**) SDS-PAGE gel from the $Yfh1^{GST}$-NIA-Isu1 pull-down experiment; position of ACP_{Ec} band is indicated (**B**) Identification of peptides detected in LC-MS/MS analysis of the excised ACP_{Ec} band was performed in PEAKS Studio software, using PEAKS standard protocol, based on *E. coli* and *S. cerevisiae* protein sequences from UniProtKB database, as described in the Experimental procedures. PEAKS significance score (-10lgP), coverage of protein sequences with peptides and number of ACP_{Ec} peptides detected are indicated.
